# Arylsulfatases and neuraminidases modulate engagement of CCR5 by chemokines by removing key electrostatic interactions

**DOI:** 10.1038/s41598-023-50944-1

**Published:** 2024-01-02

**Authors:** Inês Pinheiro, Nicolas Calo, Marianne Paolini-Bertrand, Oliver Hartley

**Affiliations:** 1 Department of Pathology and Immunology, Faculty of Medicine, University of Geneva, Geneva, Switzerland; 2 Orion Biotechnology, Campus Biotech Innovation Park, Geneva, Switzerland

**Keywords:** G protein-coupled receptors, Chemokines, Post-translational modifications

## Abstract

The chemokine receptor CCR5 is known to exist in cell surface subpopulations that differ in their capacity to engage ligands. One proposed explanation for this phenomenon is the presence of CCR5 species with different levels of post-translational modifications (PTMs). Tyrosine sulfation and O-glycan sialylation are PTMs that add negative charges to the extracellular domain of CCR5 and make strong contributions to chemokine binding but it is not known whether cellular mechanisms to control their levels exist. In this study we used a combination of sulfation-sensitive and sulfation-insensitive CCR5 ligands to show that the rate of turnover of CCR5 tyrosine sulfation is more rapid than the rate of turnover of the receptor itself. This suggests that the steady state level of CCR5 sulfation is maintained through the combination of tyrosine protein sulfotransferase (TPST), the trans-Golgi network (TGN)-resident ‘source enzyme, and a ‘sink’ activity that removes tyrosine sulfation from CCR5. By measuring the effects on ligand binding of knockdown and overexpression experiments, we provided evidence that non-lysosomal cellular arylsulfatases, particularly ARSG, ARSI and ARSJ, are CCR5 sulfation ‘sink’ enzymes. We also used targeted knockdown and sialylation-sensitive and insensitive chemokines to identify the sialidase NEU3 as a candidate ‘sink’ enzyme for CCR5 O-glycan sialylation. This study provides the first experimental evidence of activity of sulfatase and sialidase ‘sink’ enzymes on CCR5, providing a potential mechanism for cells to control steady-state levels of these PTMs and thereby exert dynamic control over receptor-ligand interactions at the cell surface and during receptor desensitization.

## Introduction

The CC chemokine receptor 5 (CCR5) is a G protein-coupled receptor (GPCR) whose main physiological role is the direction of leukocyte trafficking in response to inflammatory stimuli^1^. In addition to being the principal coreceptor for HIV^2^, CCR5 is implicated in neuroinflammatory disease^3^, virally driven acute respiratory distress syndrome (e.g. COVID-19)^4^, and cancer^5^.

The established natural chemokine ligands of CCR5 are CCL3, CCL4 and CCL5. These small proteins engage CCR5 via a “two-site” mechanism characteristic of the chemokine system^6,7^, in which the rigid core region of the chemokine binds to the receptor N-terminus and extracellular loops (CRS1), and the flexible N-terminal region of the chemokine engages the transmembrane domain of the receptor (CRS2) leading to signal transduction. The CRS1 binding component is dominated by electrostatic interactions between a positively charged groove on the chemokine core region and negative charges located on the receptor extracellular N-terminal region, with a major component of receptor negative charges provided by post-translational modifications (PTMs), including tyrosine sulfation and O-glycan sialylation.

Tyrosine sulfation has been demonstrated or predicted across many members of the chemokine receptor family^8,9^, and is catalyzed by tyrosylprotein sulfotransferases (TPSTs) that reside in the trans-Golgi network^10,11^. The N-terminal region of CCR5 has four tyrosine residues (Y3, Y10, Y14, Y15) susceptible to sulfation by TPSTs^12^, all of which have been shown to contribute to the binding affinity of native chemokines^13,14^. Sialyation of O-glycans on extracellular serine residues S6 and S7 also makes an important contribution to the binding affinity of native CCR5 ligands^15^.

Cell surface CCR5 has been shown to be comprised of subpopulations that differ (1) in their capacity to be engaged by anti-CCR5 monoclonal antibodies (mAbs)^16,17^, (2) in their function as HIV coreceptors and sensitivity to small molecule CCR5 inhibitors^18,19^, and (3) in their binding affinity for chemokines^14,20^. The origin and nature of these subpopulations remains unclear, but heterogeneity in CCR5 PTMs^18,21^ is one of the explanations that have been put forward.

Recently, mAbs directed towards the extracellular N-terminal region of CCR5 that differ in their requirements for CCR5 sulfation for binding to their epitopes were identified^14^. In addition, CCL5 analogs engineered for enhanced CRS2 interactions^22,23^ have been shown to be capable of binding a much higher proportion of the cell surface CCR5 pool than native chemokines^14,20,24^ and not to be dependent on CCR5 sulfation for receptor engagement^14^. These sulfation-sensitive and sulfation-insensitive ligands were used to demonstrate that subpopulations of CCR5 with different levels of tyrosine sulfation exist at the cell surface and that the relative level of CCR5 sulfation can be modulated by either raising or lowering the activity of TPST^14^.

In this study we made use of these sulfation-sensitive and sulfation-insensitive CCR5 ligands to investigate the origin of CCR5 sulfation heterogeneity and to explore the possibility that cells might control levels of CCR5 engagement by chemokines through a combination of enzyme activities that add or subtract PTMs carrying negative charges.

## Materials and methods

### Chemokines and antibodies

Human CCL5, 5P12-CCL5 and PSC-CCL5 used in this study were prepared by chemical synthesis as previously described^22,23^. Fluorescent derivatives of the chemokines were generated as previously described^25^, except that the fluorochrome used for derivatization was 5-(and-6)-carboxytetramethylrhodamine rather than Cy5. The following anti-CCR5 monoclonal antibodies were used: phycoerythrin (PE)-labelled mAb 3A9 (BD Biosciences Cat# 550632, RRID:AB_2072548), Alexa647-labelled rat mAb HEK/1/85a (Bio-Rad Cat# MCA2175, RRID:AB_32433).

### Plasmids

*pCLX-ARS-mirGE inducible knockdown vectors.* The microRNA-based lentivector (mirGE) inducible knockdown system^26–28^ was used to target each of the 8 ARS candidates. An automated scoring system^29^ was used to identify sequence stretches in each target ARS mRNA and design appropriate mirGE DNA sequences. Synthesized target DNA (Thermo Fisher Scientific) was cloned into an acceptor vector (pENTR) by ligation and then transferred by gateway cloning into an autoinducible lentivector plasmid (pCLX) as previously described^28^.

*pFUGW-ARS-T2A-mCherry overexpression vectors.* ARS-T2A-mCherry genes were obtained by custom gene synthesis for ARSG, ARSI and ARSJ, with DNA encoding the self-cleaving peptide T2A sequence (EGRGSLLTCGDVEENPGP) between ARS and mCherry. The open reading frames were then cloned into the FUGW lentiviral expression vector by restriction cloning (XbaI/EcoRI cloning sites).

### Cell lines

All cells in this study were maintained in DMEM supplemented with 10% FBS and 1% Penicillin/ Streptavidin (Thermo Fisher Scientific).

*HEK-CCR5 cells.* We made use of a previously described human embryonic kidney 293 T (HEK)-CCR5 clonal cell line stably expressing human CCR5^22^.

*HEK-CCR5 ARS-mirGE cells.* HEK-CCR5 cells were transduced with lentiviral particles generated using appropriate pCLX-ARS-mirGE plasmids (individually or combined for multiple knockdown, MOI = 3) and selected with blasticidin 20 μg/mL (Invivogen) for 2 weeks.

*HEK-CCR5 ARS-T2A-mCherry cells.* Cell lines were obtained by lentiviral transduction with appropriate pFUGW-ARS-T2A-mCherry plasmids followed by population selection by fluorescence-activated cell sorting (mCherry fluorescence).

### ARS and NEU3 knockdown

*ARS.* mirGE-mediated ARS knockdown in HEK-CCR5 ARS-mirGE was induced via the pTF promotor with 1 μg/ mL doxycycline (Sigma Aldrich). After 4 days of induction, cells were detached with EDTA prior to analysis.

*NEU3.* HEK-CCR5 cells were transfected with siRNA specific for the human NEU3 (Cat# SI03147655 FlexiTube siRNA), negative control siRNA (Cat# 1027292, AllStars Negative Control siRNA) and positive control siRNA (Cat# 1027298, AllStars Hs Cell Death Control siRNA) with HiPerFect transfection reagent for 72 h.

### Flow cytometry binding assays

HEK-CCR5 cells under the different conditions were detached with EDTA 0.5 mM and incubated with either 1:100 of fluorochrome-labelled mAbs or with rhodamine-labelled chemokines at 300 nM diluted in FACS buffer (1 × PBS, 1 mM EDTA, 1% BSA) in 96-well plates. Following 1 h incubation, cells were washed once in FACS buffer and ligand binding was measured by flow cytometry on a Cytoflex instrument (Beckman Coulter) with 10^4^ events collected and median fluorescence values were obtained from CytExpert (Beckman Coulter).

### Calcium flux assays

HEK-CCR5 cells were detached and seeded (2 × 10^4^ cells/well) in 384-well black and clear flat bottom plates (GREINER). 4 h later, cells were loaded with a calcium-sensitive fluorescent dye (Screen Quest™ Fluo-8 No Wash Calcium Assay Kit, AAT Bioquest, Lubio Science) according to the manufacturer’s instructions. Fluorescence signals (excitation, 490 nm; emission, 525 nm) were recorded before and after addition of agonist (dissolved in PBS supplemented with 1% BSA and 25 mM Hepes) at defined concentrations using an FDSS instrument (HAMAMATSU). Agonist responses were obtained by dividing the fluorescence signal obtained following agonist treatment by that of a control well with cells treated with buffer only and determining the peak height.

### Time course experiments with sodium chlorate and cycloheximide

HEK-CCR5 cells were seeded in 6 well-plates and incubated in sulfate-free media (DMEM/Nutrient Mixture F-12 Ham from Sigma-Aldrich) supplemented with 10% FBS with or without sodium chlorate (100 mM, Sigma-Aldrich). After 2 h, 4.5 h, 9 h and 18 h cells were detached with 0.5 mM EDTA in PBS, washed twice and incubated at 4 °C with labelled antibodies (PE-labelled 3A9 or Alexa647-labelled HEK/ 1/85a) at 1:100 dilution prior to analysis by flow cytometry.

### Sialidase treatment

HEK-CCR5 cells (1 × 10^7^) were detached with 0.5 mM EDTA in PBS, washed once with medium, and treated for 1.5 h at 37 °C with 200 μL DMEM supplemented with 0.3 U *Arthrobacter ureafaciens* sialidase from (Roche) or with 200 μL unsupplemented DMEM. Cells were then washed twice with FACS buffer (1 × PBS, 1 mM EDTA, 1% BSA) prior to analysis by flow cytometry.

### Statistical analysis

All statistical analyses were performed with GraphPad Prism 10.0 using log-transformed values for results expressed as percentages (control set as 100%). Except where indicated, differences between two groups were assessed using two-tailed parametric paired t-tests and multiple comparisons were carried out using one-way ANOVA with Dunnett’s T3 post-hoc test (conditions versus control). *p*-values of < 0.05 were considered to be statistically significant (*p < 0.05, **p < 0.01, ***p < 0.001).

## Results

### Evidence for rapid turnover of cellular CCR5 sulfation

**Figure 1:**
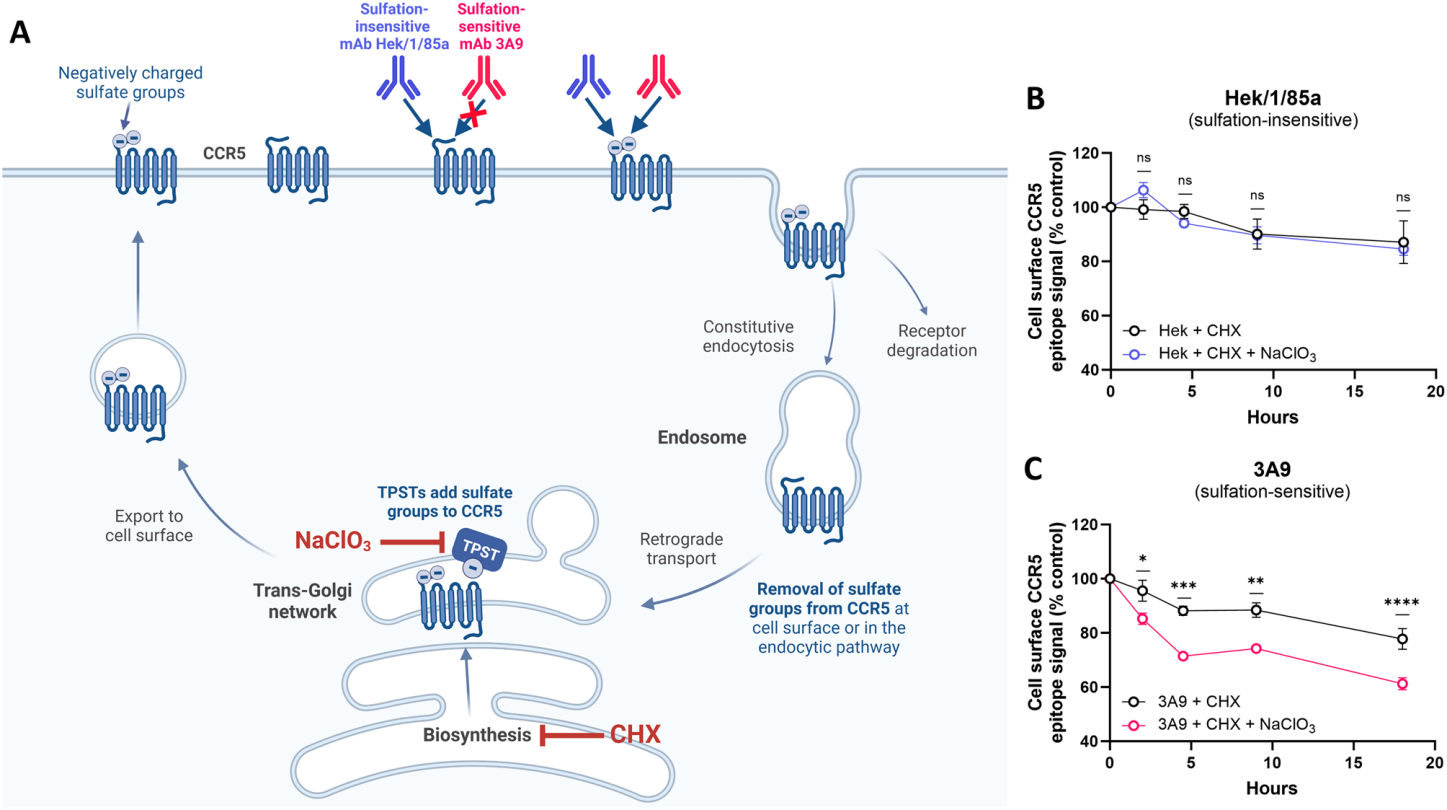
Rapid turnover of tyrosine degradation as CCR5 cycles through the cell. (**A**) CCR5 cycles spontaneously through the cell in a process of endocytosis followed by recycling to the cell surface via the TGN. HEK-CCR5 cells were treated with cycloheximide (CHX) to prevent newly biosynthesized CCR5 from entering the cellular CCR5 pool, and receptor turnover was measured using sulfation-sensitive (3A9) and sulfation-insensitive (Hek/1/85a) anti-CCR5 mAbs in the presence or absence of TPST blockade using sodium chlorate. Created with Biorender. (**B**) Time-course determination of cell surface CCR5 on HEK-CCR5 cells using flow cytometry with anti-CCR5 sulfation-insensitive mAb Hek/1/85a and (**C**) sulfation-sensitive anti-CCR5 mAb 3A9. Binding signals are expressed as % control (CTRL) $\left[{(\mathrm{median\,\, fluorescence\,\, intensity }({\text{MFI}})}_{\mathrm{CHX \,\,or\,\, CHX}+{\text{NaClO}}_3 })/{MFI}_{CTRL}\times 100\right]$, where CTRL corresponds to sulfate-free medium without inhibitors. Data show mean binding signals ± SEM from 3 independent experiments. 2-way ANOVA analysis was performed on measurements made at each timepoint using log-transformed values.

Exploiting the phenomenon that chemokine receptors including CCR5 undergo spontaneous endocytosis and recycling in the absence of agonist activation^30–32^, and excluding de novo biosynthesized CCR5 from the total receptor pool by treating cells with cycloheximide, we used sulfation-sensitive (mAb 3A9) and sulfation-insensitive (mAb Hek/1/85a) anti-CCR5 mAbs^14^ to determine the effects of TPST blockade on levels of cell surface CCR5 sulfation (Fig. 1). Cycloheximide treatment alone (Fig. 1B,C and Fig. S1) reduced binding signals of both sulfation-sensitive and sulfation-insensitive anti-CCR5 mAbs to a comparable extent, with a rate of epitope removal (approximately 20% over the 18 h measurement period) indicative of slow receptor turnover, as previously observed for both CCR5^33^ and CCR7^34^. Chlorate is a competitive inhibitor of ATP sulfurylase that blocks the production of 3’-phosphoadenosine-5’-phosphosulfate, the sulfate donor coenzyme used by TPST^35^. Treatment of cells with sodium chlorate in addition to cycloheximide did not change either the extent or the rate of binding signal decay for the sulfation-insensitive mAb Hek/1/85a (Fig. 1B), but led to a substantial reduction in the binding signal for the sulfation-sensitive mAb 3A9 (Fig. 1C). This implies that the degradation rate for CCR5 tyrosine sulfation is considerably more rapid than that of the cellular pool of CCR5, and that in the absence of inhibition, CCR5 sulfation is replenished by TPST as the receptor cycles through the TGN^25,36^.

### Arylsulfatases: candidate cellular enzymes for driving CCR5 tyrosine sulfation turnover

**Figure 2:**
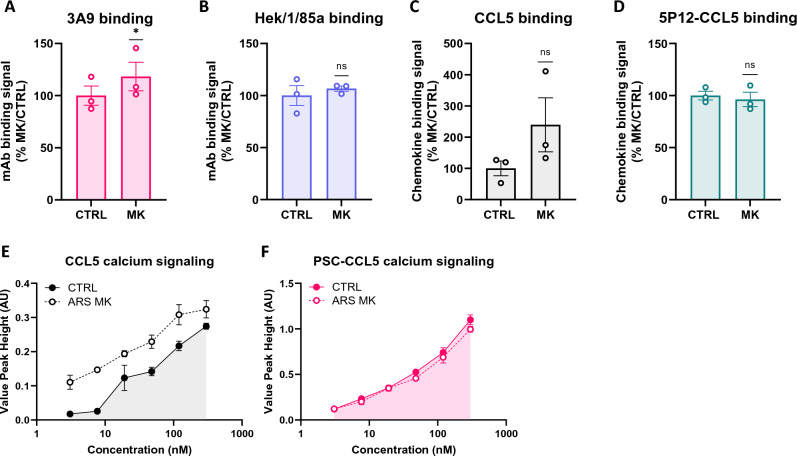
Multiple knockdown of ARS enzymes leads to increased engagement of sulfation-sensitive CCR5 ligands. (**A**–**D**) HEK-CCR5 cells in which multiple ARS MirGE knockdown was induced (MK) or uninduced (CTRL) were incubated at 4 °C for 1h with sulfation-sensitive mAb 3A9 (**A**) and insensitive mAb Hek/1/85a (**B**), 300 nM rhodamine-labelled CCL5 (**C**) or 300 nM rhodamine-labelled 5P12-CCL5 (**D**). Binding signals are expressed as % CTRL $\left[{(MFI}_{ARS MK}-cells \,\,autofluorescence\,\, (AF))/{(MFI}_{CTRL}-AF)\times 100\right]$. Data represent 3 independent experiments and p-values were calculated using a paired t-test (uninduced versus induced for each experimental replicate) on log-transformed values. (**E**, **F**) CTRL and MK cells were stimulated with CCL5 (**E**) or PSC-CCL5 (**F**) at the indicated concentrations and calcium flux signals were measured. Data points represent mean ± SEM of triplicate peak heights and are representative of 3 independent experiments.

Since sulfotyrosines are chemically stable under conditions corresponding to the cellular environment^37^, we hypothesized that turnover of CCR5 tyrosine sulfation might occur as a consequence of a cellular sulfatase enzyme activity. Using a previously described microRNA-based inducible knockdown strategy^26,27^, we simultaneously targeted all non-lysosomal members (ARS C through J) of the arylsulfatase family^38,39^, and measured the consequences for engagement of sulfation-sensitive and sulfation-insensitive CCR5 ligands (Fig. 2).

Multiple knockdown of arylsulfatases (Fig. S2) led to an increased level of binding of the sulfation-sensitive anti-CCR5 mAb 3A9 (18% increase, p = 0.0194) without affecting that of the sulfation-insensitive mAb Hek/1/85a (Fig. 2A,B and Fig. S3A). It led to a substantial, albeit not statistically significant, increase in the level of binding of native CCL5, but had no effect on that of 5P12-CCL5 (Fig. 2C,D, respectively and Fig. S3B), an engineered CCL5 antagonist analog^23^ with increased CCR5 binding capacity (Fig. S4) previously shown to bind to CCR5 in a sulfation-insensitive manner^14^. It also led to an increase in the CCR5 signaling activity of CCL5 (Fig. 2E and Fig. S5A), but not that of the engineered superagonist PSC-CCL5 (Fig. 2F and Fig. S5B), which binds to and activates a larger population of cell surface CCR5 than CCL5 (^20,24^ Fig. S4 and S6).

These results show that reducing the cellular expression of non-lysosomal arylsulfatases leads to an increase in the capacity of sulfation-sensitive CCR5 ligands to engage CCR5, supporting a role for these enzymes in CCR5 tyrosine sulfation turnover.

### Individual ARS knockdown reveals ARSI, ARSJ and ARSG as candidate enzymes for driving CCR5 tyrosine sulfation turnover

**Figure 3:**
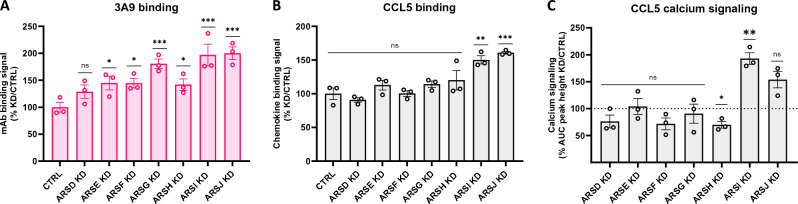
Effect of individual knockdown of arylsulfatases on the engagement of sulfation-sensitive CCR5 ligands. (**A**, **B**) HEK-CCR5 parental (CTRL) or ARS MirGE induced individual knockdown (KD) cells were incubated at 4°C for 1h with mAb 3A9 (**A**) or 300 nM rhodamine-labelled CCL5 (**B**). Binding signals are expressed as % CTRL $\left[{(MFI}_{ARS KD}-AF)/{(MFI}_{CTRL}-AF)\times 100\right]$. Data represent 3 independent experiments and p-values were calculated using a one-way ANOVA on log-transformed values. (**C**) HEK-CCR5 cells in which ARS MirGE individual knockdown was induced (KD) or uninduced (CTRL) were stimulated with serial dilutions of CCL5 (300, 120, 48, 19) and calcium flux signals were measured (dotted line corresponds to the average of each uninduced CTRL samples, set as 100%). Data are expressed as % CTRL $\left[{(\mathrm{AUC \,\,of\,\, dose\,\, response\,\, peak\,\, height}}_{ARS\,\, KD}/{AUC}_{CTRL})\times 100\right]$. Data represent 3 independent experiments and p-values were calculated using a paired t-test (uninduced versus induced for each experimental replicate) on log-transformed values.

In order to determine which of the eight ARS candidates might be responsible for driving CCR5 sulfation turnover, we used the same microRNA-based inducible knockdown strategy to perform individual knockdown of the non-lysosomal arysulfatases, opting to exclude ARSC which has a well-characterized substrate specificity for sulfated steroids^40,41^. We then measured the effects of individual knockdown on the binding of sulfation-sensitive anti-CCR5 mAb 3A9 as well as the binding and signaling activity of sulfation-sensitive native CCL5 (Fig. 3).

Individual knockdown (Fig. S7) led to increased binding of sulfation-sensitive anti-CCR5 mAb 3A9 of between 30 and 100% across the panel of arylsulfatases, with the largest effects seen with ARSG (80%, *p* = 0.0006), ARSI (97%, p = 0.0002) and ARSJ (100%, *p* = 0.0001) (Fig. 3A and Fig. S8A). Individual knockdown of ARSI and ARSJ also led to statistically significant increases in the binding of native CCL5 (50%, *p* = 0.0033 and 61%, p = 0.0008, respectively) (Fig. 3B and Fig. S8B), as well as in its signaling activity (93%, *p* = 0.0069 and 54%, p = 0.0589 respectively) (Fig. 3C and Fig. S9). Hence individual knockdown of either ARSI or ARSJ is sufficient to recapitulate the cellular phenotype observed with the ARS multiple knockdown (Fig. 2). Since ARSG gave a very strong expression signal in HEK cells (Fig. S2A) and targeting of ARSG led to a pronounced increase in sulfation-sensitive antibody binding (Fig. 3A) despite the relatively low-level of knockdown achieved in the experiment (Fig. S7), we opted to perform further studies on ARSG in addition to ARSI and ARSJ.

### Overexpression of ARSG, ARSI and ARSJ reduces the capacity of sulfation-sensitive ligands to engage CCR5

**Figure 4:**
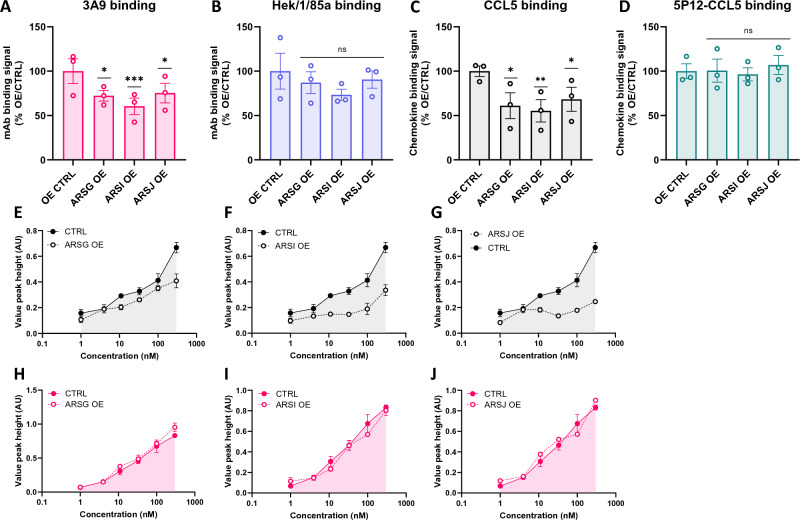
Overexpression of ARSG, ARSI, and ARSJ leads to decreased engagement of sulfation-sensitive CCR5 ligands. (**A**–**D**) HEK-CCR5 parental cells (CTRL) or cells stably transduced with the indicated FUGW-ARS-T2A-mCherry constructs (ARS OE) were incubated at 4 °C for 1h with mAb 3A9 (**A**) and mAb Hek/1/85a (**B**), 300 nM rhodamine-labelled CCL5 (**C**) and rhodamine-labelled 5P12-CCL5 (**D**). Binding signals are expressed as % CTRL $\left[{(MFI}_{ARS OE}-AF)/{(MFI}_{CTRL}-AF)\times 100\right]$. Data represent 3 independent experiments and p-values were calculated using a one-way ANOVA using log-transformed values. (**E–J**) CTRL and ARS OE cells were stimulated with CCL5 (**E**–**G**) or PSC-CCL5 (**H**–**J**) at the indicated concentrations and calcium flux signals were measured. Data points represent mean peak height of triplicates ± SEM and data are representative of independent experiments (3 for CCL5, 2 for PSC-CCL5).

We next used ARS-T2A-mCherry plasmids to individually overexpress ARSG, ARSI and ARSJ (Fig. S10) and measured its effect on the binding and signaling of sulfation-sensitive and insensitive CCR5 ligands (Fig. 4). Overexpression of all three arylsulfatases led to decreases in the binding levels of the anti-CCR5 mAbs, but in each case there was a greater impact on the binding of the sulfation-sensitive mAb 3A9 (28%, *p* = 0.0120; 40%, *p* = 0.0010; 25%, *p* = 0.0178, respectively for ARSG, ARSI and ARSJ) compared to that of the sulfation-insensitive mAb Hek/1/85a (Fig. 4A,B, respectively and Fig. S11). Native CCL5 binding was also decreased for each arylsulfatase (39%, *p* = 0.0148; 45%, *p* = 0.0071; 32%, *p* = 0.0469 respectively for ARSG, ARSI and ARSJ), with binding of 5P12-CCL5 unaffected (Fig. 4C,D, respectively and Fig. S12). The signaling activity of native CCL5 was also decreased for each arylsulfatase, while there was no effect on signaling of PSC-CCL5 (Fig. 4C–H and Fig. S13). Together, these results provide further support for a role of candidate arylsulfatases ARSI, ARSJ and ARSG in driving the turnover of CCR5 sulfation.

### A possible role for cellular NEU3 sialidase in driving CCR5 O-glycan sialylation turnover

**Figure 5:**
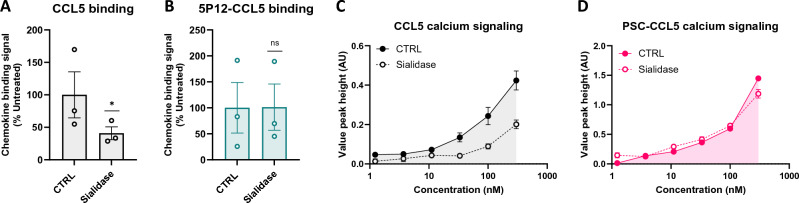
Removal of sialic acids from cell surface glycoproteins including CCR5 leads to a decrease in the binding capacity of native chemokines. (**A**, **B**) Sialidase-treated (sialidase) and untreated (CTRL) HEK-CCR5 and HEK-WT cells were incubated at 4 °C for 1h with rhodamine-labelled chemokine CCL5 (**A**) and 5P12-CCL5 (B) at 300 nM. Specific binding signals are expressed as percentage of untreated cells corrected to HEK-WT $\left[{(MFI}_{{\text{HEK}}-{\text{CCR}}5\mathrm{ CTRL\,\, or\,\, sialidase}}-AF)/{(MFI}_{{\text{HEK}}-\mathrm{WT\,\, CTRL\,\, or\,\, sialidase }}-AF)\times 100\right]$. Data represent 3 independent experiments and p-values were calculated using a paired t-test (untreated versus treated for each experimental replicate) on log-transformed values. (**C**, **D**) Sialidase-treated and untreated HEK-CCR5 cells were stimulated with CCL5 or PSC-CCL5 at the indicated concentrations and calcium flux signals were measured. Data points represent mean peak height of triplicates ± SEM and data are representative of 3 independent experiments.

To investigate whether CCR5 sialylation might be subjected to cellular turnover in a manner analogous to that of CCR5 sulfation, we began by treating HEK-CCR5 cells with exogenous *Arthrobacter ureafaciens* sialidase, whose efficient removal of CCR5 sialylation was confirmed by detecting a quantitative electrophoretic mobility shift in Western blot (Fig. S14A), and observing the effects on the binding of native CCL5 and 5P12-CCL5. In line with previous observations, we observed an almost complete reduction in the binding capacity of native CCL5 (59%, *p* = 0.0176) upon sialidase treatment^15^, whereas the binding capacity of 5P12-CCL5 was not affected (Fig. 5A,B, respectively and Fig. S14B). Similarly, the CCR5 signaling activity of native CCL5 was substantially reduced, in contrast to that of PSC-CCL5 which was unchanged (Fig. 5C,D, respectively and Fig. S14C).

**Figure 6:**
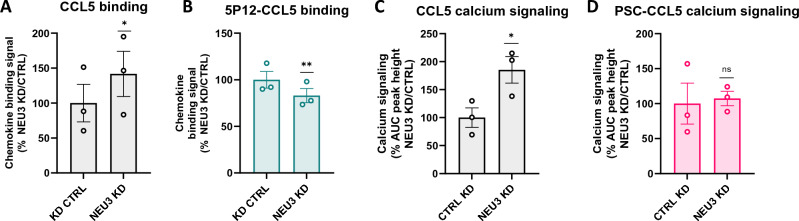
NEU3 knockdown leads to increased binding and signaling activity of native CCL5. (**A**, **B**) HEK-CCR5 cells transfected with NEU3 siRNA (KD) or negative control siRNA (CTRL) were incubated at 4 °C for 1h with 300 nM rhodamine-labelled chemokines CCL5 (**A**) and 5P12-CCL5 (**B**). Binding signals are expressed as % CTRL WT $\left[{(MFI}_{{\text{NEU}}3 KD}-AF)/{(MFI}_{{\text{CTRL}}}-AF)\times 100\right]$. (**C**, **D**) HEK-CCR5 NEU3 KD and CTRL cells were stimulated with serial dilutions of CCL5 (**C**) or PSC-CCL5 (**D**) (300, 120, 48, 19, 8, 3 nM) and calcium flux signals were measured. Data are expressed as % CTRL $\left[{(\mathrm{AUC\,\, of\,\, dose\,\, response\,\, peak\,\, height}}_{{\text{NEU}}3\mathrm{ KD}})/{AUC}_{\mathrm{CTRL }})\times 100\right]$. (**A**–**D**) data represent 3 independent experiments and *p*-values were calculated using a paired t-test (control versus knockdown for each experimental replicate) on log-transformed values.

Having determined that engineered CCL5 analogs can be used as sialylation-insensitive probes, we next investigated the potential involvement of the endosome-resident sialidase NEU3^42,43^ in CCR5 sialylation turnover. Knockdown of NEU3 using siRNA (Fig. S15) led to an increase in binding of CCL5 (42%, *p* = 0.0413) while decreasing the binding of 5P12-CCL5 to a small (17%) but nonetheless statistically significant (*p* = 0.0029) extent (Fig. 6A,B, respectively and Fig. S16A). In addition, it led to an 85% increase (*p* = 0.0102) in the CCR5 signaling activity of CCL5 but did not affect that of PSC-CCL5 (Fig. 6C,D and Fig. S16B and C). These results provide evidence that cellular CCR5 sialylation levels are modulated in a manner analogous to that of CCR5 sulfation as the receptor cycles through the cell, with NEU3 sialidase playing a potential role in CCR5 desialylation during endocytosis.

## Discussion

In this study, we used previously characterized sulfation-sensitive and sulfation-insensitive CCR5 ligands^14^ to investigate the origins of CCR5 sulfation heterogeneity and the potential modulation of CCR5 cell surface PTMs that carry negative charges. Our observation that turnover of sulfated CCR5 at the cell surface takes place at a considerably faster rate than turnover of the total CCR5 pool when TPST activity is blocked (Fig. 1), led us to infer that in the absence of inhibition, TPST contributes to the maintenance of steady-state levels of CCR5 sulfation, replenishing sulfation that is lost at the cell surface or during transit through the cell. Given the relatively high chemical stability of the tyrosine sulfoester bond^40^, we postulated that CCR5 sulfation turnover is driven enzymatically by one or more cellular arylsulfatases. In support of this idea, we observed that multiple knockdown of the non-lysosomal arysulfatases ARS C through J increases steady-state levels of sulfated CCR5 at the cell surface (Fig. 2), an effect that was recapitulated with individual knockdown of the ARS genes, particularly ARSG, ARSI and ARSJ (Fig. 3). Correspondingly, overexpression of ARSG, ARSI and ARSJ reduced steady-state levels of CCR5 sulfation at the cell surface (Fig. 4).

These observations suggest that cell surface CCR5 sulfation heterogeneity is established by the relative contributions of the sulfation ‘source’ (TPST) and the sulfation ‘sink’ enzyme activities (ARS). The work in this study confirms and extends earlier observations^14^ to show that it is possible to raise or lower steady-state cell surface CCR5 sulfation levels by interfering with expression levels of the ‘source’ and ‘sink’ enzymes. Such modulation would alter the responsiveness of cells to native chemokines, which require CCR5 sulfation for high affinity binding^15,21,46^. Indeed, we showed that modulation of the ‘sink’ enzymes for CCR5 sulfation affects both the binding capacity and the signaling activity of native chemokines (Figs. 3, 4 and 5). These effects are not seen with the CCL5 analogs, 5P12-CCL5 and PSC-CCL5 (Figs. 3, 4 and 5), whose lack of dependence on CCR5 sulfation for receptor engagement is likely due to enhanced interactions at CRS2 that compensate for the reduced interaction at CRS1.

Having identified candidate arylsulfatases that remove native charges from CCR5 as it traffics through the cell, we investigated whether a parallel mechanism might exist for the removal of sialic acids from CCR5. We confirmed the previous observation that engagement of CCR5 by native CCL5 is sensitive to the sialyation state of the receptor^15^, noting that the N-terminally modified CCL5 analogs 5P12-CCL5 and PSC-CCL5 are insensitive (Fig. 5). Again, this lack of sensitivity is likely due to compensation for the weakened electrostatic interaction at CRS1 caused by removal of sialic acid residues on the receptor by enhanced CRS2 interactions.

Emerging evidence suggests that sialyl-O-glycosylation and tyrosine sulfation work in a coordinated manner as ‘source’ enzymes that add negative charges to proteins in the secretory pathway^21,44^, including chemokine and chemokine-like receptors^9,15,45^. We investigated NEU3 as a candidate ‘sink’ enzyme for CCR5 sialylation because it is the only one of the four known mammalian sialidases that is localized in both endosomal structures and at the cell surface^46^, and which has been implicated in clathrin-mediated endocytosis^42,43^. Knockdown of NEU3 led to an increase in the relative binding level of native CCL5 compared to that of 5P12-CCL5 (Fig. 6A,B) and an increase in the relative signaling level of native CCL5 compared to that of PSC-CCL5 (Fig. 6C,D). These observations provide a preliminary indication that levels of CCR5 sialylation could be controlled by a combination of the activity of ‘source’ enzyme sialyltransferases (STs) in the TGN and ‘sink’ enzyme sialidases in the endocytic pathway.

Further work will be required to confirm and consolidate the observations we have made. First, it will be necessary to replicate the key findings of this study on primary cells that endogenously express CCR5. Second, while we were able to benefit from sulfation-sensitive and sulfation-insensitive anti-CCR5 mAbs to measure CCR5 sulfation levels, a better understanding of CCR5 sulfation heterogeneity could be attained by quantification of sulfation levels at each implicated tyrosine residue, for example using mass spectrometry-based approaches such as those recently introduced to measure protein sialylation patterns^47^. Finally, we used knockdown and overexpression experiments to implicate at least three different arylsulfatases in CCR5 desulfation and NEU3 as a candidate enzyme for CCR5 desialylation. Further work will be required to confirm a role for these enzymes in this process by analogy with work that has been done to characterize TPSTs^10–12^, including (1) determining the subcellular location of these relatively uncharacterized enzymes, and (2) showing that the enzymes are capable of using synthetic CCR5 peptide derivatives as substrates.

In summary, our findings provide a first indication that cells have a mechanism to modulate their response to chemokines by dynamically shaping levels of tyrosine sulfation and O-glycan sialylation on CCR5. Such a control mechanism, exerted by modifying activity of the of the ‘source’ and ‘sink’ enzymes at the transcriptional level or via direct activation or inhibition mechanisms, could provide a system to fine-tune immune responses mediated by CCR5. Dysregulation in the expression and/or activity of these enzymes could also have pathological consequences, and indeed overexpression of NEU3 has been linked to inflammatory diseases such as intestinal inflammation and colitis^48^, and aberrant expression of both NEU3^49^ and ARSI^50^ in tumors has been linked to cancer progression.

In addition, dynamic control of CCR5 tyrosine sulfation and O-glycan sialylation could help elucidate a component of the CCR5 desensitization and resensitization process that is not currently understood. It is known that natural chemokines dissociate from CCR5 during desensitization^33,51^, but this cannot be explained by exposure to low pH in the endosomal pathway^52^ as has been indicated for other GPCRs^53^. It is possible that arylsulfatases and sialidases resident in the endocytic pathway could drive chemokine dissociation by removing essential CCR5 negative charges at CRS1. Subsequent cycling of desensitized CCR5 via the TGN would then enable resident TPSTs and STs to replenish the CRS1 negative charges so that CCR5 is returned to the cell surface in a form capable of being engaged by chemokines.

Importantly, these dynamic mechanisms could be extended to other receptors in the chemokine family, the majority of which have been shown to or predicted to have sulfation and O-glycosylation sites in their extracellular N-terminal domains^8,9^.

### Supplementary Information

Supplementary Information.

## Data Availability

This study includes no data deposited in external repositories.
