# Author Correction: Arylsulfatases and neuraminidases modulate engagement of CCR5 by chemokines by removing key electrostatic interactions

**DOI:** 10.1038/s41598-024-57806-4

**Published:** 2024-03-28

**Authors:** Inês Pinheiro, Nicolas Calo, Marianne Paolini-Bertrand, Oliver Hartley

**Affiliations:** 1 Department of Pathology and Immunology, Faculty of Medicine, University of Geneva, Geneva, Switzerland; 2 Orion Biotechnology, Campus Biotech Innovation Park, Geneva, Switzerland

Correction to: *Scientific Reports* 10.1038/s41598-023-50944-1, published online 02 January 2024

The original version of this Article contained an error in Figure 1, panel B, where the label “Hek/1/85a (sulfation-insensitive)” was incorrectly given as “Hek/1/85a (sulfation-sensitive)”. The original Figure 1 and accompanying legend appear below.

**Figure 1:**
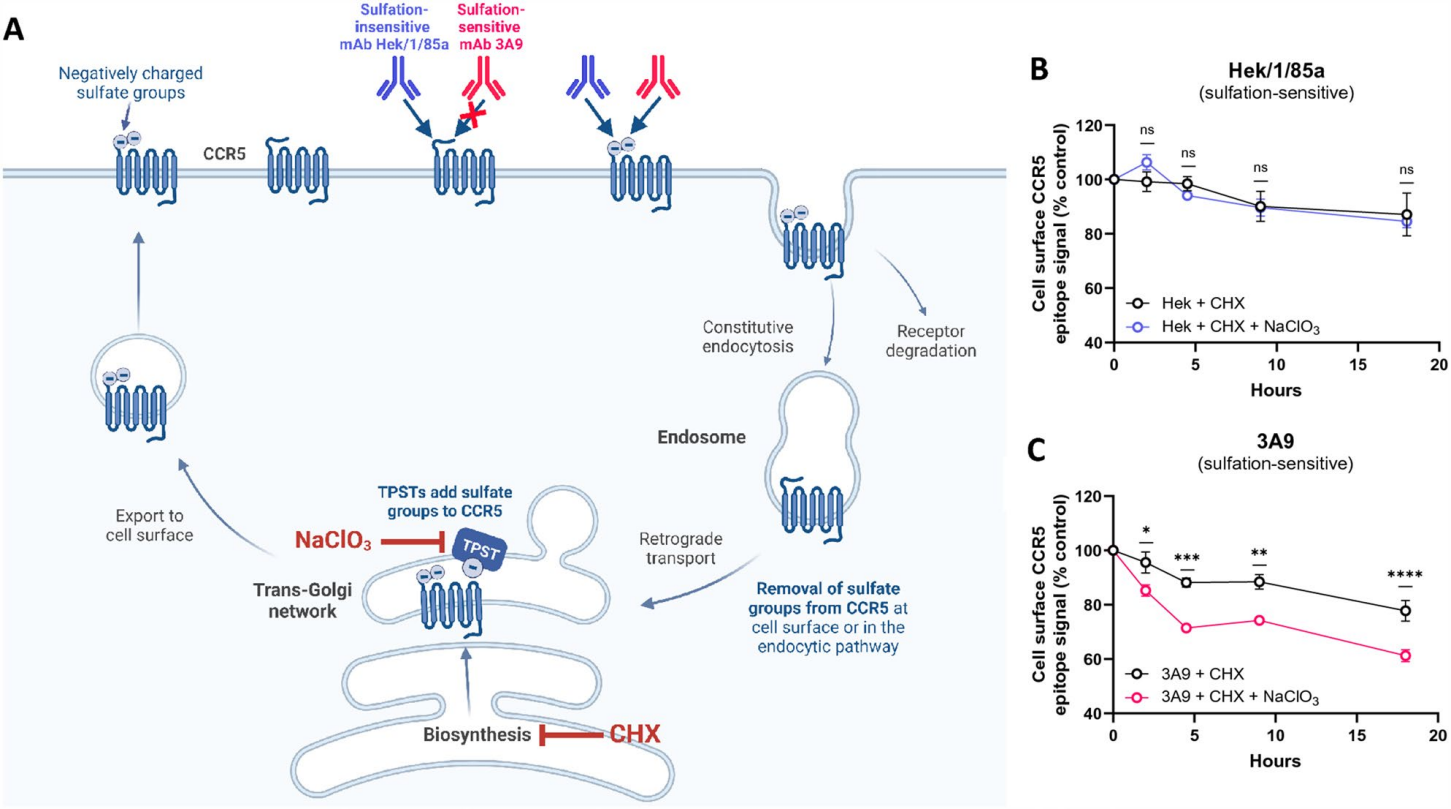
Rapid turnover of tyrosine degradation as CCR5 cycles through the cell. (**A**) CCR5 cycles spontaneously through the cell in a process of endocytosis followed by recycling to the cell surface via the TGN. HEK-CCR5 cells were treated with cycloheximide (CHX) to prevent newly biosynthesized CCR5 from entering the cellular CCR5 pool, and receptor turnover was measured using sulfation-sensitive (3A9) and sulfation-insensitive (Hek/1/85a) anti-CCR5 mAbs in the presence or absence of TPST blockade using sodium chlorate. Created with Biorender. (**B**) Time-course determination of cell surface CCR5 on HEK-CCR5 cells using flow cytometry with anti-CCR5 sulfation-insensitive mAb Hek/1/85a and (**C**) sulfation-sensitive anti-CCR5 mAb 3A9. Binding signals are expressed as % control (CTRL) $\left[ {({\text{median fluorescence intensity}}\left( {{\text{MFI}}} \right)_{{{\text{CHX or CHX}} + {\text{NaClO}}_{3} }} } \right)/MFI_{CTRL} \times 100]$, where CTRL corresponds to sulfate-free medium without inhibitors. Data show mean binding signals ± SEM from 3 independent experiments. 2-way ANOVA analysis was performed on measurements made at each timepoint using log-transformed values.

The original Article has been corrected.

