# Supplementary material for: Arylsulfatases and neuraminidases modulate engagement of CCR5 by chemokines by removing key electrostatic interactions
Source: Sci Rep. 2024 Jan 2;14:292. doi: 10.1038/s41598-023-50944-1 (PMC10762049; doi:10.1038/s41598-023-50944-1)
Supplement: Supplementary file 1 — Supplementary Information. [file 41598_2023_50944_MOESM1_ESM.pdf]

# Supplementary material

## Arylsulfatases and neuraminidases modulate engagement of CCR5 by chemokines by removing key electrostatic interactions

Scientific Reports

Inês Pinheiro[1], Nicolas Calo [1,2], Marianne Paolini-Bertrand [1], Oliver Hartley [1,2]

[1] Department of Pathology and Immunology, Faculty of Medicine, University of Geneva, Switzerland

[2]Orion Biotechnology, Campus Biotech Innovation Park, Geneva, Switzerland

**Corresponding author:**

Oliver Hartley, Oliver.Hartley@unige.ch

## Supplementary Materials and Methods

### Western Blotting

HEK-CCR5 and HEK-WT cells were cultivated to semi-confluency (approximately 8–10 million cells) in 10 cm Petri dishes. Cells were treated for 1.5 h at 37°C with 200 µL DMEM supplemented with 0.3 U *Arthrobacter ureafaciens* sialidase from (Roche) or with 200 µL unsupplemented DMEM. After, cells were resuspended in 100 µL of lysis buffer (Tris 50 mM, 1% NP40 supplemented with Halt™ Protease inhibitor 1× (Thermo Fisher Scientific) and left on ice for 40 min at 4°C. Lysis supernatant was centrifuged for 10 min at 14000 rpm in a bench top centrifuge and lysates were dosed for protein content using Pierce™ BCA Protein Assay Kit (Thermo Fisher Scientific). Samples corresponding to HEK-WT, untreated HEK-CCR5 and HEK-CCR5 sialidase treated, were mixed with NuPAGE LDS Sample Buffer (Invitrogen) and DTT (final concentration of 0.1 M), heated to 100°C for 3 min and loaded on a 15-lane Nu-PAGE™ 4–12% Bis-Tris gel (Invitrogen) prior to electrophoresis in MES SDS running buffer (Novex®) for 35 min at 200 V. Samples were then transferred on to nitrocellulose membranes using iBlot® 2NC Regular stacks (Invitrogen). Membranes were blocked at room temperature for 1 h in PBS-3% BSA-0.1% Tween (PBS-BSA-T) and after washed 3 times in PBS-BSA-T. Then membranes were incubated at room temperature for 1 h with 2 µg/mL primary antibody anti-CCR5 rabbit ab63123 polyclonal antibody (Abcam Cat# ab63123, RRID:AB_1139407) in PBS-BSA-T. Membranes were washed once in PBS-BSA-T (10 min on rocker) and twice in PBS-T (no BSA) (2 x 10 min on rocker), then incubated for at room temperature for 1 h with 1:2000 of secondary antibody goat anti-rabbit HRP (Agilent Cat# P0448, RRID:AB_2617138) in PBS-BSA-T. After one washing step in PBS-BSA-T (10 min) and two washing steps in PBS-T (15 min), membranes were developed using WesternBright™ Quantum western blotting detection kit (1:1).

## Quantitative reverse transcription PCR (RT-qPCR)

Total RNA prepared from HEK-CCR5 that had been subjected to the different knockdown and overexpression conditions was extracted using a Monarch® Total RNA Miniprep Kit (New England Biolabs) and RNA quality was verified using the RNA 6000 Nano Kit (Agilent). cDNA was synthesized from 0.5 µg of total RNA using a mix of random hexamers and oligo d(T) primers and PrimerScript reverse transcriptase enzyme (Takara) according to the manufacturer's instructions. SYBR green assays were designed using Primer Express v2.0 software (Applied Biosystems) with default parameters. Custom primers (**Table S1**) were obtained from Thermo Fisher Scientific and PCR was performed on an SDS 7900 HT instrument (Applied Biosystems), with the following parameters: 50°C for two minutes, 95°C for 10 min, and 40 cycles of 95°C 15 seconds followed by 60°C 1 min. Each reaction was performed in three replicates on 384-well plate. Ct values were obtained using SDS 2.2 software (Applied Biosystems) and imported in Excel and fold changes were calculated using the delta Ct method (Yuan et al., 2006).

**Table S1.** Custom-designed gene-specific primers for RT-qPCR

| Gene | Primers |
|---|---|
| ARSC | Forward: TGAGCCCACTAGCAACATGG |
| | Reverse: CCAGCCAGCTTGGCTACTGTA |
| ARSD | Forward: CCCTGAACCACGGATTTGAC |
| | Reverse: TTGTGAGCGTGAAGGGCAT |
| ARSE | Forward: GCCCTCACACTGGTAGCAGG |
| | Reverse: CCACGAGACGGGTATCAGGT |
| ARSF | Forward: CAGCTCTGGCTCTGTGTGCA |
| | Reverse: GGTTAGGGTGAGGATGGCAAT |
| ARSG | Forward: TCTCTAGTCGTGGCTGCCGT |
| | Reverse: GGTGAAGGCAGGATTCCGAT |
| ARSH | Forward: TGCCTACAACCTGAACCGTG |
| | Reverse: AGACCACCTGACCCACCAAG |
| ARSI | Forward: ACCAGGCTAATGTCCCAACG |
| | Reverse: GGACAGTTTTCTCCCTCCCC |
| ARSJ | Forward: ATATGGTCGGAAAATGGCACTT |
| | Reverse: TGGGCATGCATTCTTTTCTG |
| NEU3 | Forward: GGCTTGTTTGGGTGTTTGTT |
| | Reverse: TTTTGAATTGGCTTGGGTTC |
| House keeping KIF11 | Forward: GCCAAACTGGCACTGGAAAA |
| | Reverse: AGGGATCCTCTTCCCAGGTA |

**Flow cytometry supplementary data**

Raw flow cytometry data from .fcs files was imported into R and processed using the openCyto and flowStats packages (lymphGate function on FSC-A, SSC-A channels, and gate_singlet function on FSC-A/H and SSC-A/H channels). Density plots were made using the geom_density function (default parameters) from the tidyverse/ggplot2 package, scaling the maximum value of each density curve to 1.

## Supplementary Results

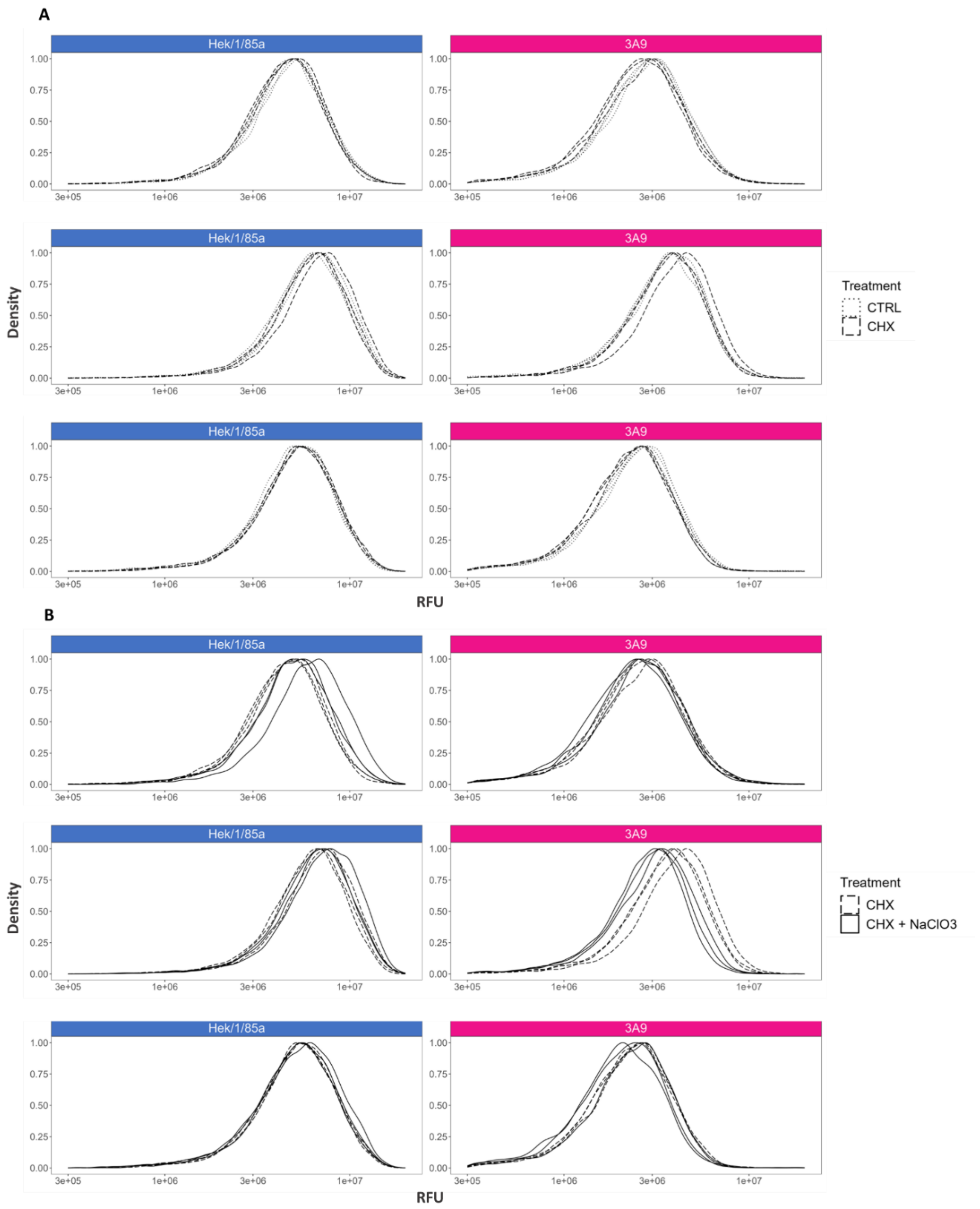


**Figure S1. A and B Time-course flow cytometry binding experiments using-sulfation-insensitive Hek/1/85a and sulfation-sensitive 3A9 anti-CCR5 mAbs.**

HEK cells cultured in sulfate-free medium supplemented by cycloheximide (CHX) 100 µg/mL in the presence or absence of sodium chlorate ($NaClO_3$) were labeled with the indicated mAbs. Control (CTRL) corresponds to signal obtained with sulfate-free medium alone. Histograms are shown for time point 2 h of 3 independent experiments. RFU = relative fluorescence units.

**C**

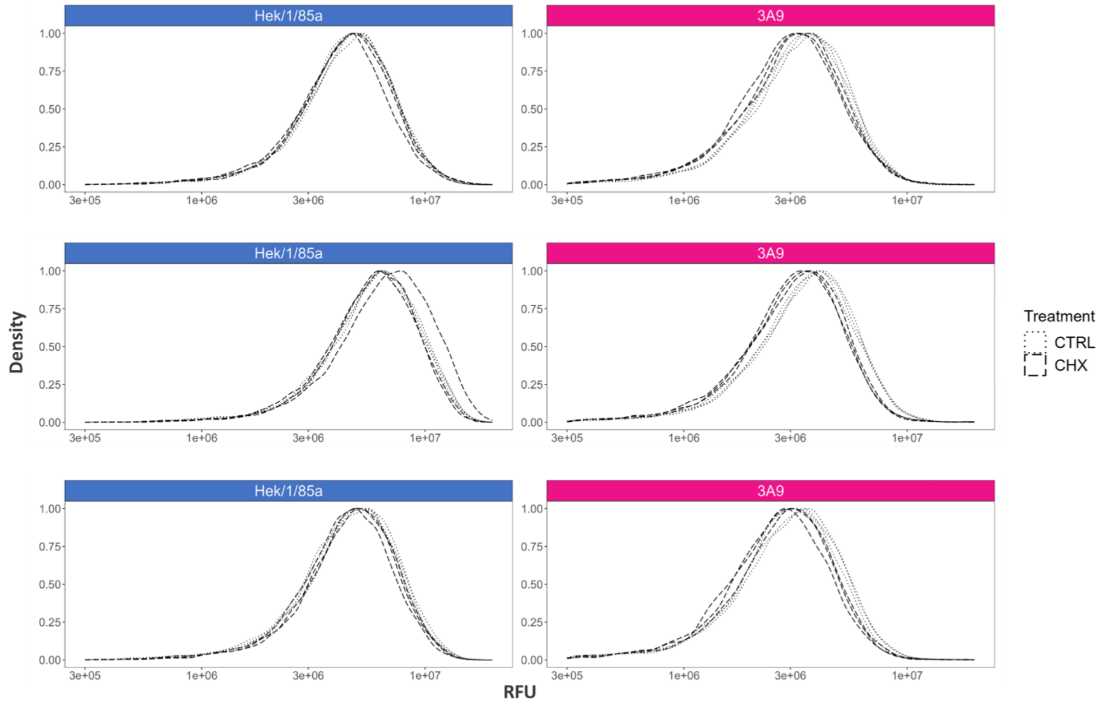


**D**

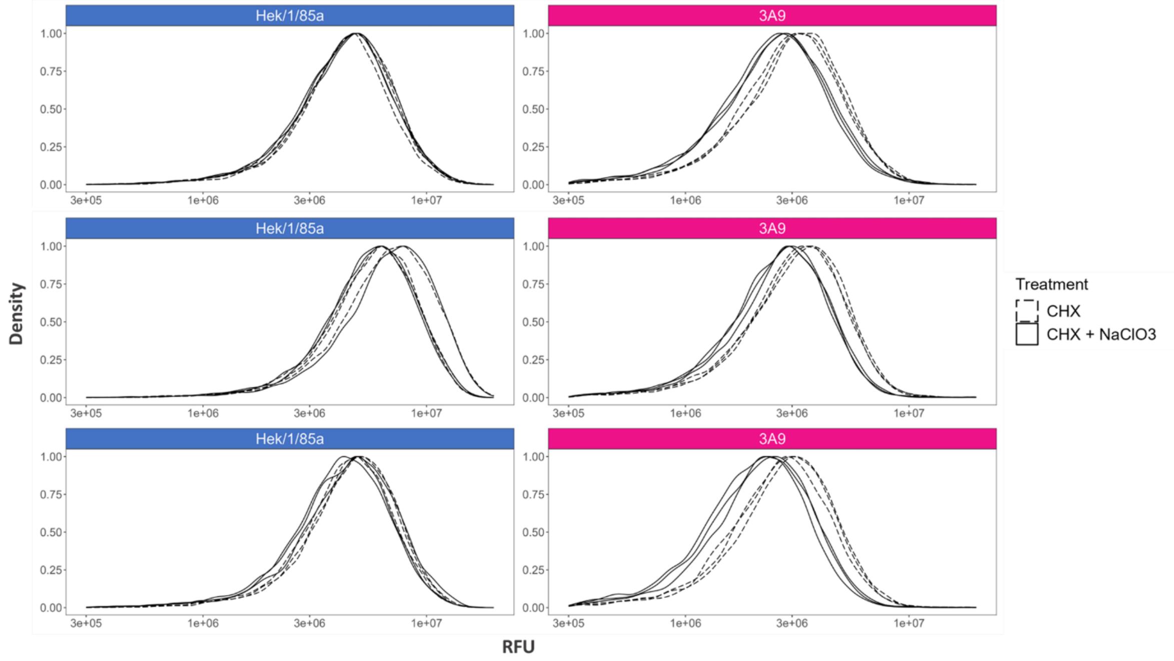


**Figure S1 (continued). C and D Time-course flow cytometry binding experiments using-sulfation-insensitive Hek/1/85a and sulfation-sensitive 3A9 anti-CCR5 mAbs.**

HEK cells cultured in sulfate-free medium supplemented by cycloheximide (CHX) 100 µg/mL in the presence or absence of sodium chlorate ($NaClO_3$) were labeled with the indicated mAbs. Control (CTRL) corresponds to signal obtained with sulfate-free medium alone. Histograms are shown for time point 4.5 h of 3 independent experiments. RFU = relative fluorescence units.

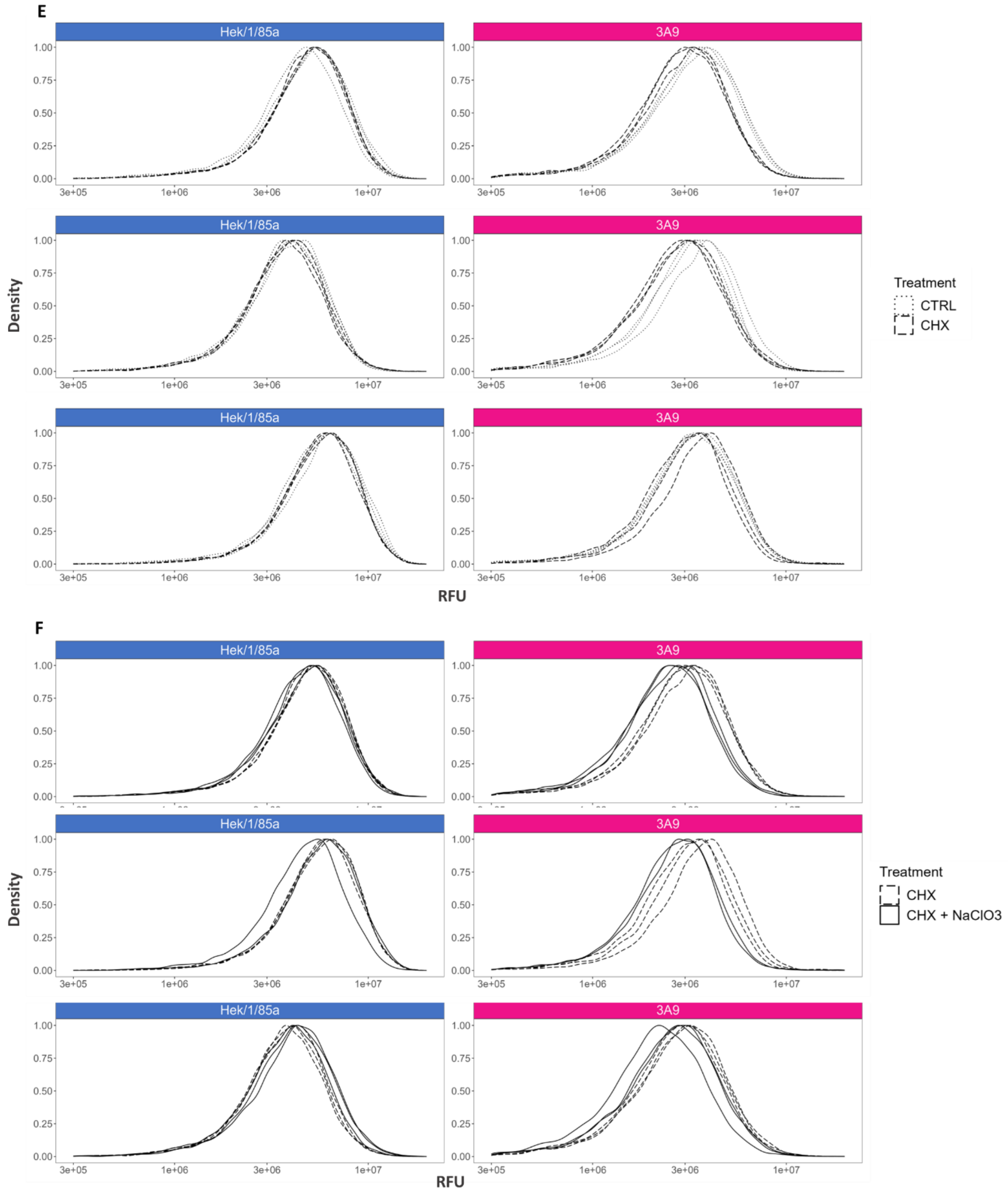


**Figure S1 (continued). E and F Time-course flow cytometry binding experiments using-sulfation-insensitive Hek/1/85a and sulfation-sensitive 3A9 anti-CCR5 mAbs.**

HEK cells cultured in sulfate-free medium supplemented by cycloheximide (CHX) 100 µg/mL in the presence or absence of sodium chlorate ($NaClO_3$) were labeled with the indicated mAbs. Control (CTRL) corresponds to signal obtained with sulfate-free medium alone. Histograms are shown for time point 9 h of 3 independent experiments. RFU = relative fluorescence units.

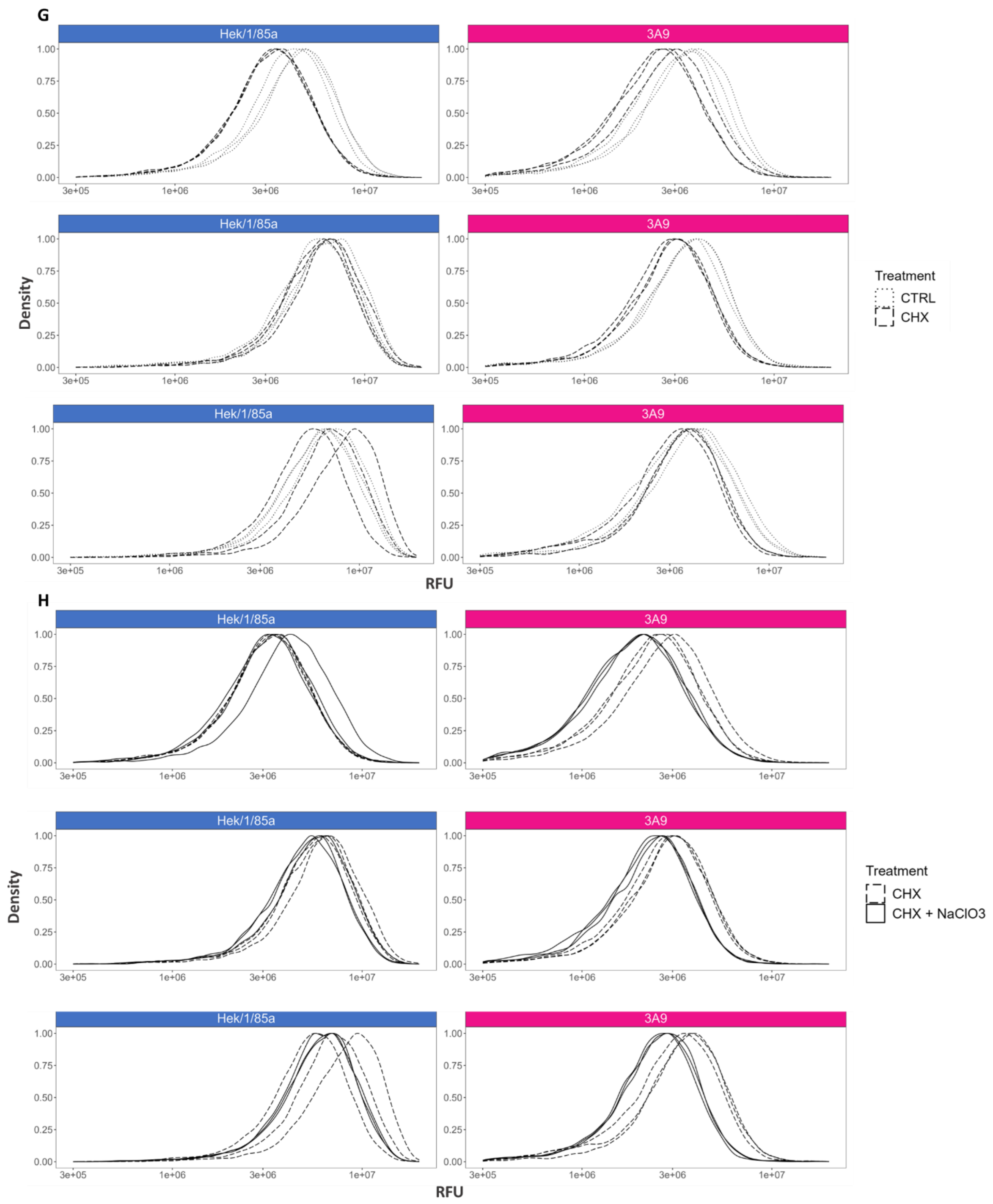


**Figure S1 (continued). G and H Time-course flow cytometry binding experiments using-sulfation-insensitive Hek/1/85a and sulfation-sensitive 3A9 anti-CCR5 mAbs.**

HEK cells cultured in sulfate-free medium supplemented by cycloheximide (CHX) 100 µg/mL in the presence or absence of sodium chlorate ($NaClO_3$) were labeled with the indicated mAbs. Control (CTRL) corresponds to signal obtained with sulfate-free medium alone. Histograms are shown for time point 18 h of 3 independent experiments. RFU = relative fluorescence units.

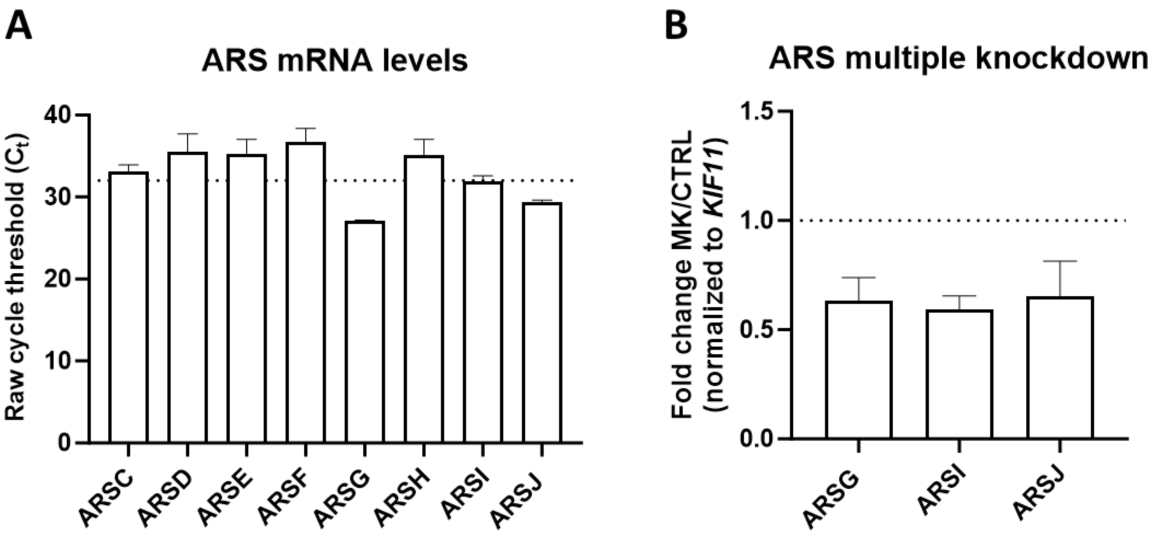


**Figure S2. Efficiency of ARS knockdown in multiple knockdown experiments.**

**A** The mRNA expression levels of the 8 ARS candidates (ARSC-J) were determined by RTqPCR on HEK-CCR5 expressing cells. Data represent the raw cycle threshold values (Cts) (mean ± SEM) of 2 independent experiments. Dashed line represents Ct=32, defined as the late cycle value near the test's limit of detection. **B** HEK-CCR5 multiple ARS MirGE knockdown uninduced cells (CTRL) and HEK-CCR5 multiple ARS MirGE knockdown induced cells (MK) mRNA levels for ARSG, I, J were assessed by RTqPCR. Data represent the fold change ratio to CTRL $[2^\wedge - \mathrm{dCt}\ (\mathrm{Ct_{ARS}} - \mathrm{Average\ Ct_{KIF11}})KD/2^\wedge - \mathrm{dCt}\ (\mathrm{Ct_{ARS}} - \mathrm{Average\ Ct_{KIF11}})CTRL]$, of 3 independent experiments (mean ± SEM). Dotted line corresponds to CTRL (set as 1). Knockdown efficiency was not calculated for ARSC D, E, F and H because initial mRNA expression levels were close to the limit of detection of the assay (cycle threshold values above 32).

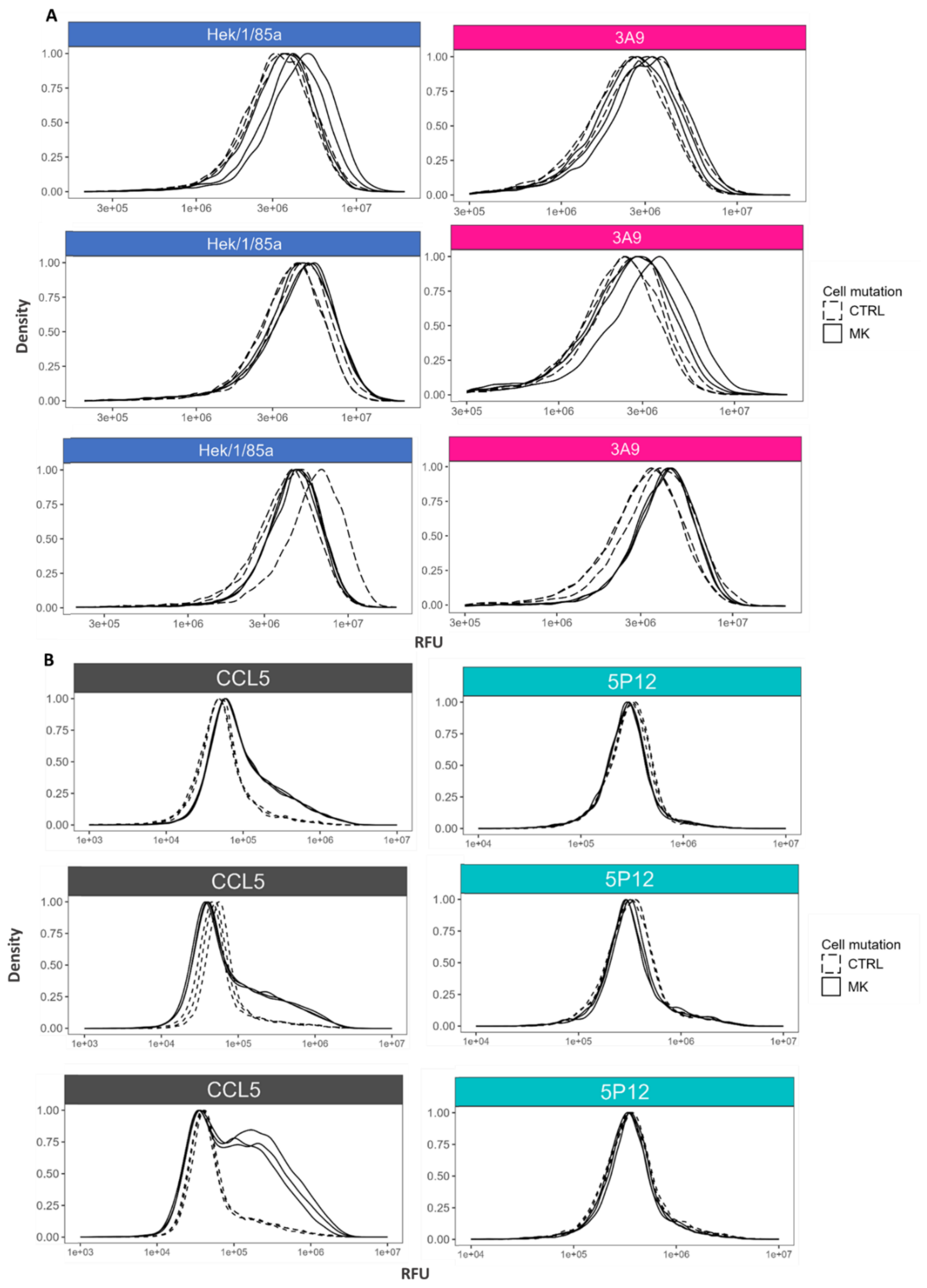


**Figure S3. Effects of ARS multiple knockdown on the binding of CCL5 and 5P12-CCL5**

**A** HEK-CCR5 multiple ARS MirGE knockdown uninduced cells (CTRL) and HEK-CCR5 multiple ARS MirGE knockdown induced cells (MK) were incubated at 4°C for 1h with sulfation-insensitive Hek/1/85a and sulfation-sensitive 3A9 anti-CCR5 mAbs. **B** CTRL and MK cells were incubated at 4°C for 1h with rhodamine-labelled chemokine CCL5 and 5P12-CCL5 at 300nM. Histograms of 3 independent experiments are shown. RFU = relative fluorescence units.

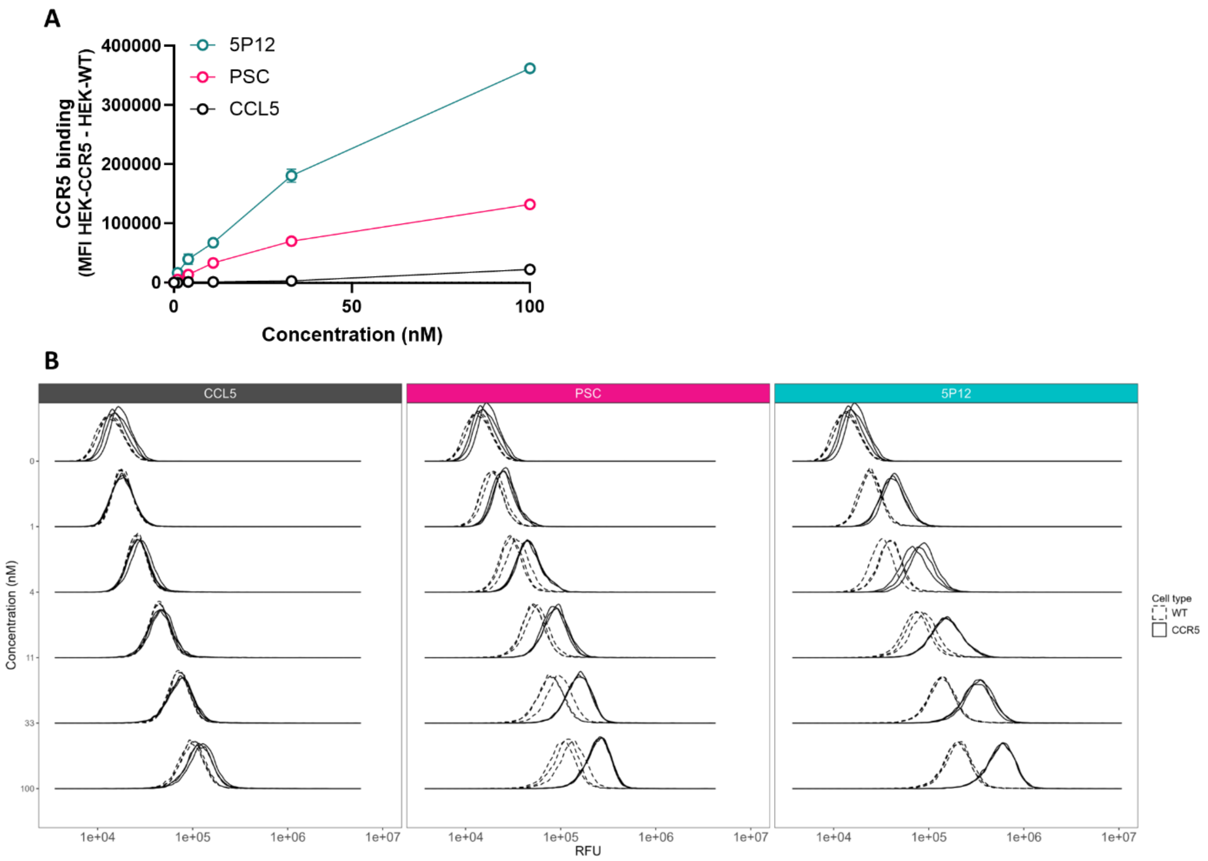


**Figure S4. 5P12-CCL5 and PSC-CCL5 show enhanced CCR5 binding capacity compared to CCL5**

**A** Flow cytometry-based saturation binding experiment using fluorescent chemokines. HEK-CCR5 and HEK-WT cells were incubated at 4°C for 1h with the indicated rhodamine-labelled chemokines CCL5, 5P12-CCL5 and PSC-CCL5. Signals were measured in the PE channel and are expressed as specific CCR5 binding signals $\mathrm{MFI}_{\mathrm{HEK-CCR5}} - \mathrm{MFI}_{\mathrm{HEK-WT}}$. Data represent mean binding signal of triplicates ± SEM and are representative of six independent experiments. **B** Representative histograms of saturation binding studies on HEK-CCR5 and HEK-WT cells with rhodamine-labelled chemokines.

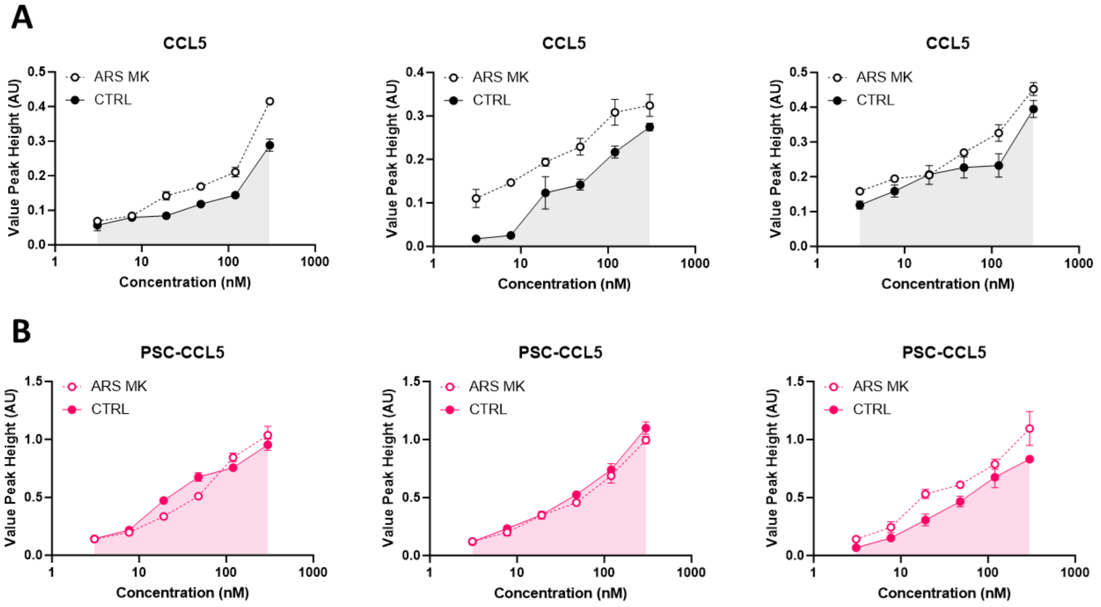


**Figure S5. Unlike CCL5, signaling of PSC-CCL5 is not affected by ARS multiple knockdown**

**A and B** HEK-CCR5 multiple ARS MirGE knockdown uninduced cells (CTRL) and HEK-CCR5 multiple ARS MirGE knockdown induced (MK) cells were incubated with CCL5 (**A**) or PSC-CCL5 (**B**) at serial dilutions (300, 100, 33, 11, 4, 1) and calcium flux signals were measured. Data points represent mean peak height of triplicates ± SEM and represent 3 independent experiments.

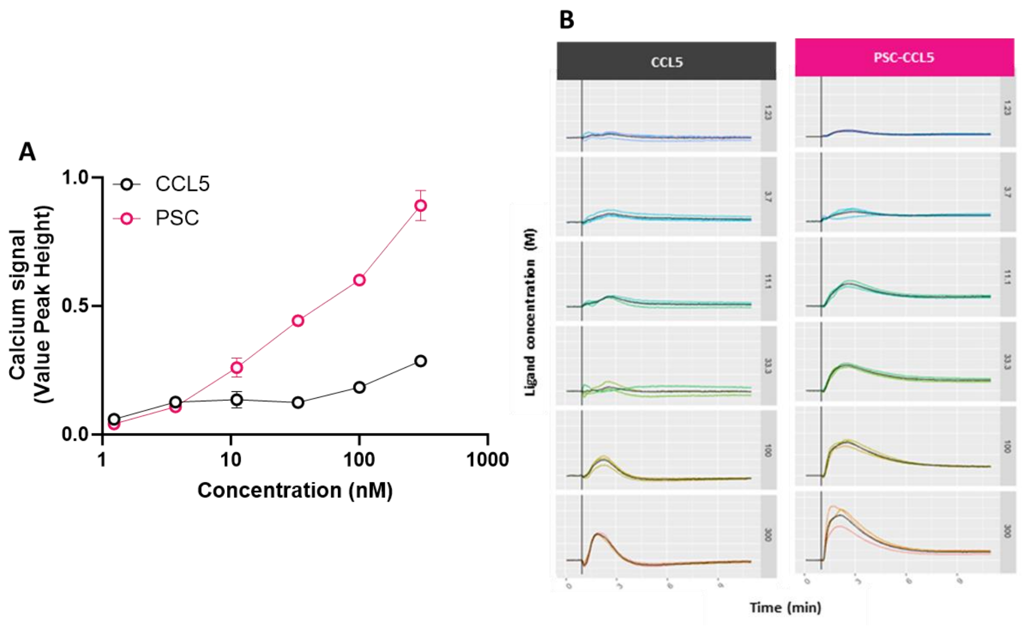


**Figure S6. PSC-CCL5 is a CCR5 superagonist**

**A** HEK-CCR5 cells were incubated with CCL5 or PSC-CCL5 at serial dilutions (300, 100, 33, 11, 4, 1) and calcium flux signals were measured. Data points represent mean peak height of triplicates ± SEM and are representative of six independent experiments. **B** Raw calcium flux data normalized to baseline and blank.

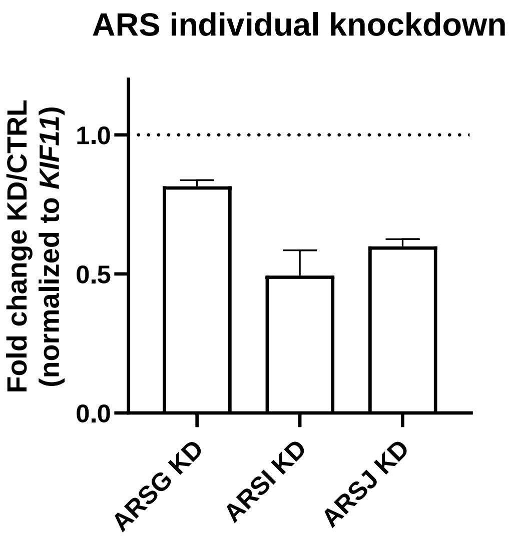


**Figure S7. Efficiency of ARS knockdown in individual knockdown experiments**

mRNA levels in HEK-CCR5 ARSG, ARSI, and ARSJ MirGE knockdown cells either uninduced (CTRL) or induced (KD) were assessed by RT-qPCR. Data represent the fold change ratio to CTRL $[2\wedge - \mathrm{dCt}\ (\mathrm{Ct}_{\mathrm{ARS}} - \mathrm{Average\ Ct}_{\mathrm{KIF11}})KD/2\wedge - \mathrm{dCt}\ (\mathrm{Ct}_{\mathrm{ARS}} - \mathrm{Average\ Ct}_{\mathrm{KIF11}})CTRL]$ from 3 independent experiments (mean ± SEM). Dotted line corresponds to CTRL (set as 1). Knockdown efficiency was not calculated for ARSD, E, F and H because initial mRNA expression levels were close to the limit of detection of the assay (cycle threshold values above 32).

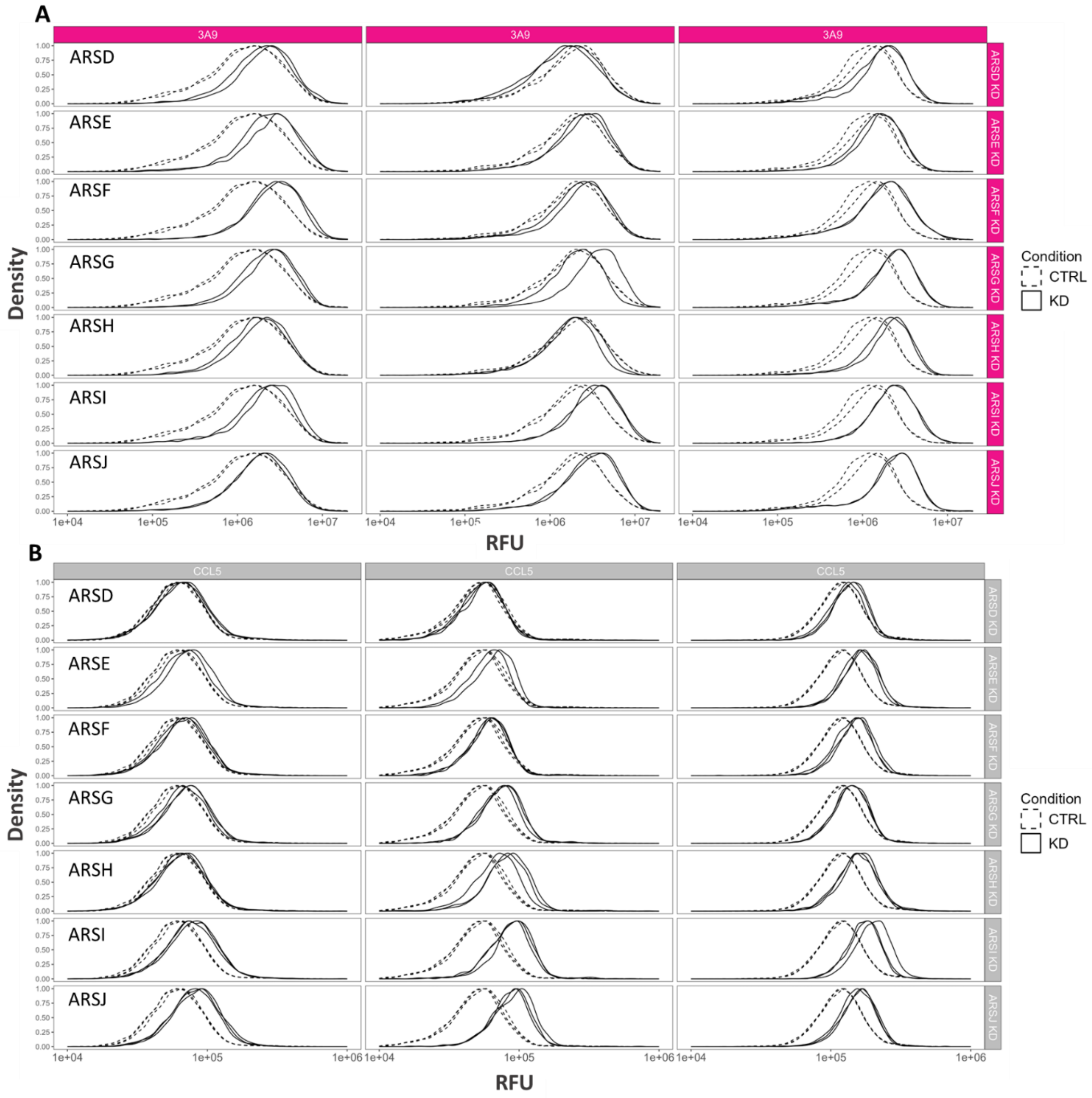


**Figure S8. Effects of individual ARS knockdown on binding of sulfation-sensitive ligands**

**A** HEK-CCR5 parental (CTRL) or ARS MirGE induced individual knockdown (KD) cells were incubated at 4°C for 1h with sulfation-sensitive mAb 3A9. **B** CTRL and KD cells were incubated at 4°C for 1h with sulfation-sensitive rhodamine-labelled chemokine CCL5 at 300nM. Histograms from 3 independent experiments are shown. RFU = relative fluorescence units.

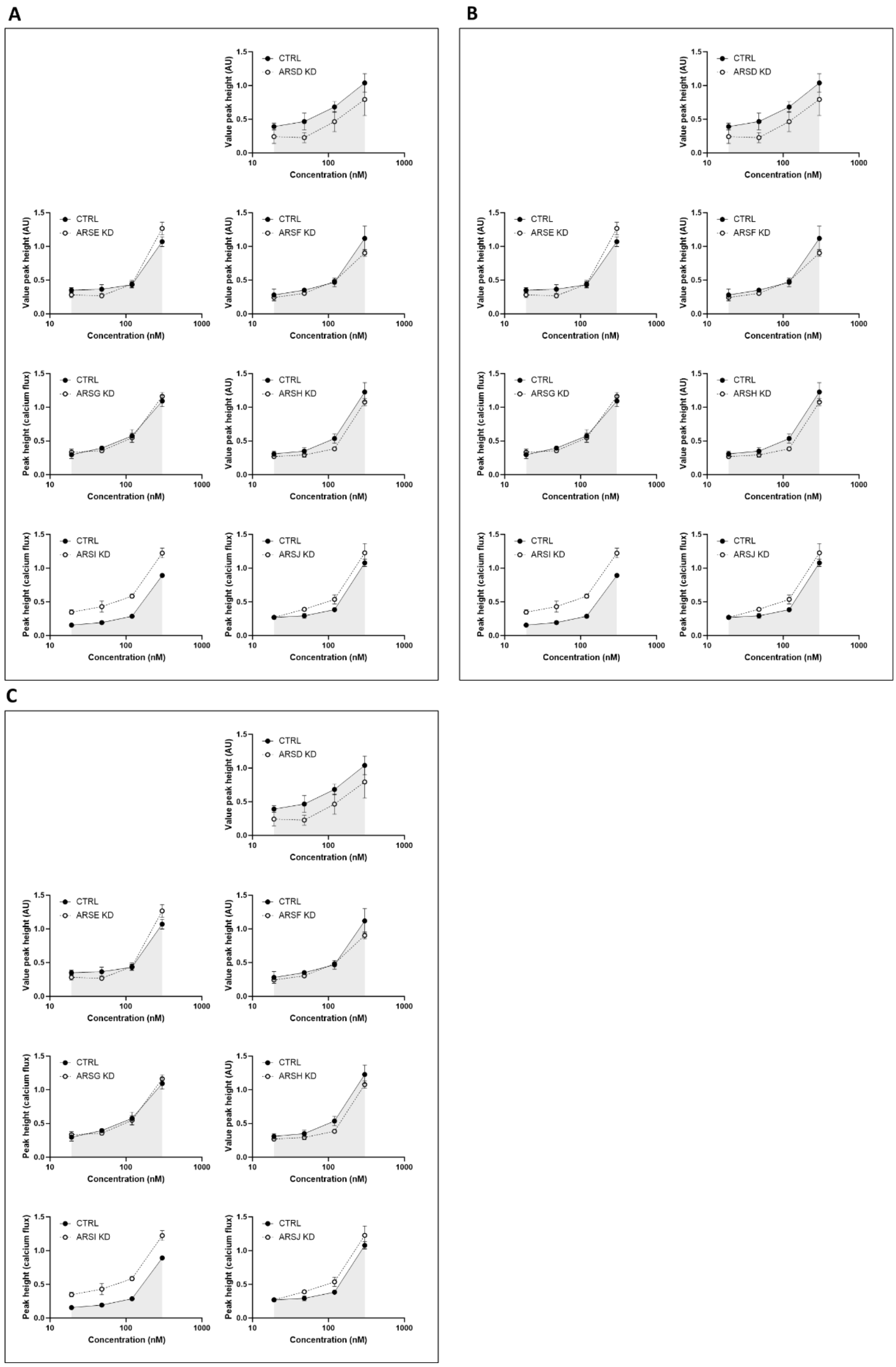


**Figure S9. Effects of individual ARS knockdown on CCL5 signaling**

**A to C** HEK-CCR5 cells in which ARS MirGE individual knockdown was induced (KD) or uninduced (CTRL) were stimulated with CCL5 at the indicated concentrations and calcium flux signals were measured. Data points represent mean peak height of triplicates ± SEM and represent 3 independent experiments (respectively **A, B, C**).

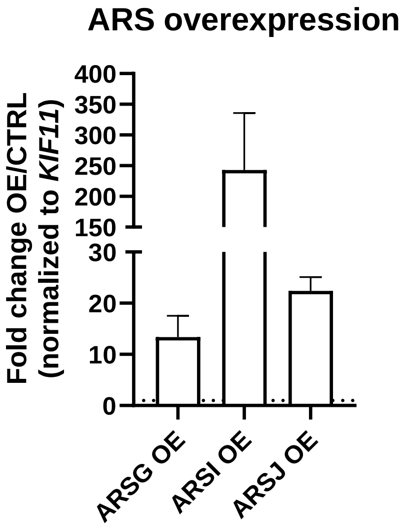


**Figure S10. Quantification of ARS overexpression**

HEK-CCR5 parental (CTRL) and HEK-CCR5 cells stably transduced with FUGW-ARS-T2A-mCherry (OE) mRNA levels were assessed by RT-qPCR. Data represent the fold change ratio to CTRL $[2^\wedge - \mathrm{dCt}\ (\mathrm{Ct}_{\mathrm{ARS}} - \mathrm{Average\ Ct}_{\mathrm{KIF11}})OE/2^\wedge - \mathrm{dCt}\ (\mathrm{Ct}_{\mathrm{ARS}} - \mathrm{Average\ Ct}_{\mathrm{KIF11}})CTRL]$ and represents 2 independent experiments (mean ± SEM). Dotted line corresponds to CTRL (set as 1).

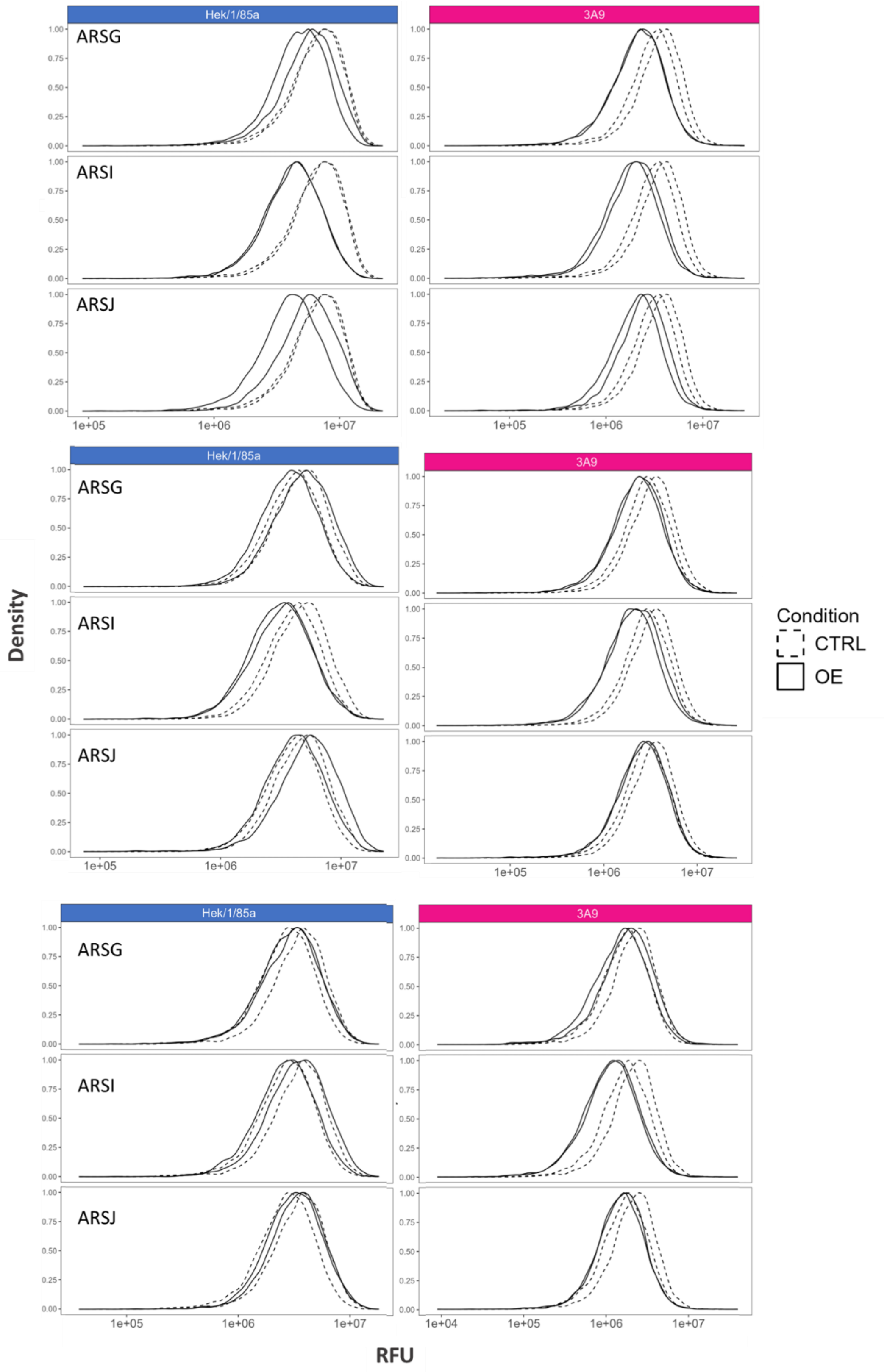


**Figure S11. Effects of ARS overexpression on binding of sulfation-sensitive and sulfation-insensitive mAbs**

HEK-CCR5 parental (CTRL) and HEK-CCR5 cells stably transduced with ARS-T2A-mCherry (OE) were incubated at 4°C for 1h with sulfation-insensitive Hek/1/85a and sulfation-sensitive 3A9 anti-CCR5 mAbs Histograms of 3 independent experiments are shown. RFU = relative fluorescence units.

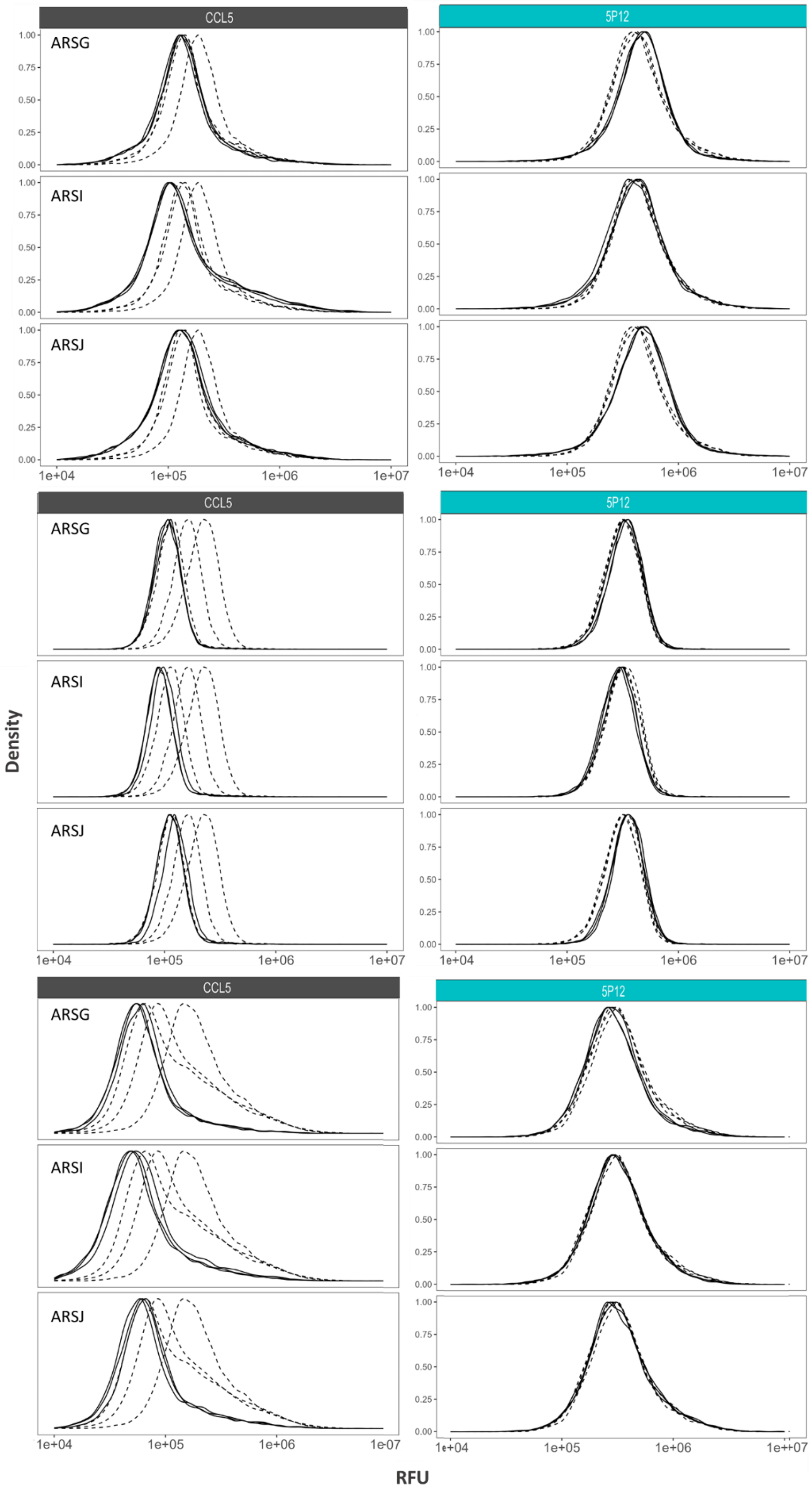


**Figure S12. Effects of ARS overexpression on binding of sulfation-sensitive and sulfation-insensitive chemokines**

HEK-CCR5 parental (CTRL) and HEK-CCR5 cells stably transduced with FUGW- ARS-T2A-mCherry cells were incubated at 4°C for 1h with rhodamine-labelled chemokine CCL5 and 5P12-CCL5 at 300nM. Histograms of 3 independent experiments are shown. RFU = relative fluorescence units.

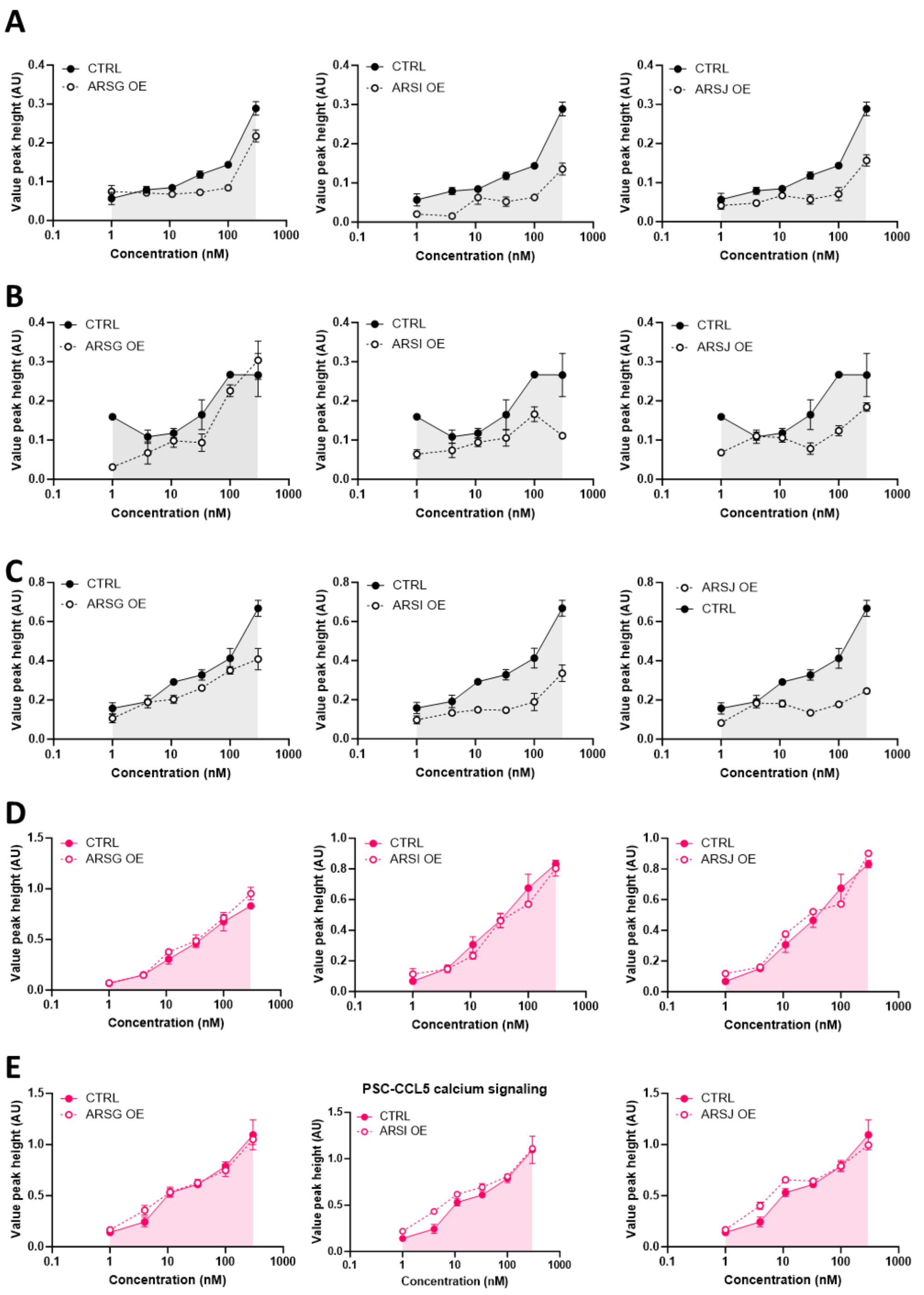


**Figure S13. Effects of ARS overexpression on signaling of sulfation-sensitive and sulfation-insensitive agonists** HEK-CCR5 parental (CTRL) and HEK-CCR5 cells stably transduced with FUGW-ARS-T2A-mCherry (OE) cells were incubated with CCL5 (**A-C**) or PSC-CCL5 (**D-E**) at the indicated concentrations and calcium flux signals were measured. Data points represent mean peak height of triplicates ± SEM and data represent 3 and 2 independent experiments (each line is one experiment).

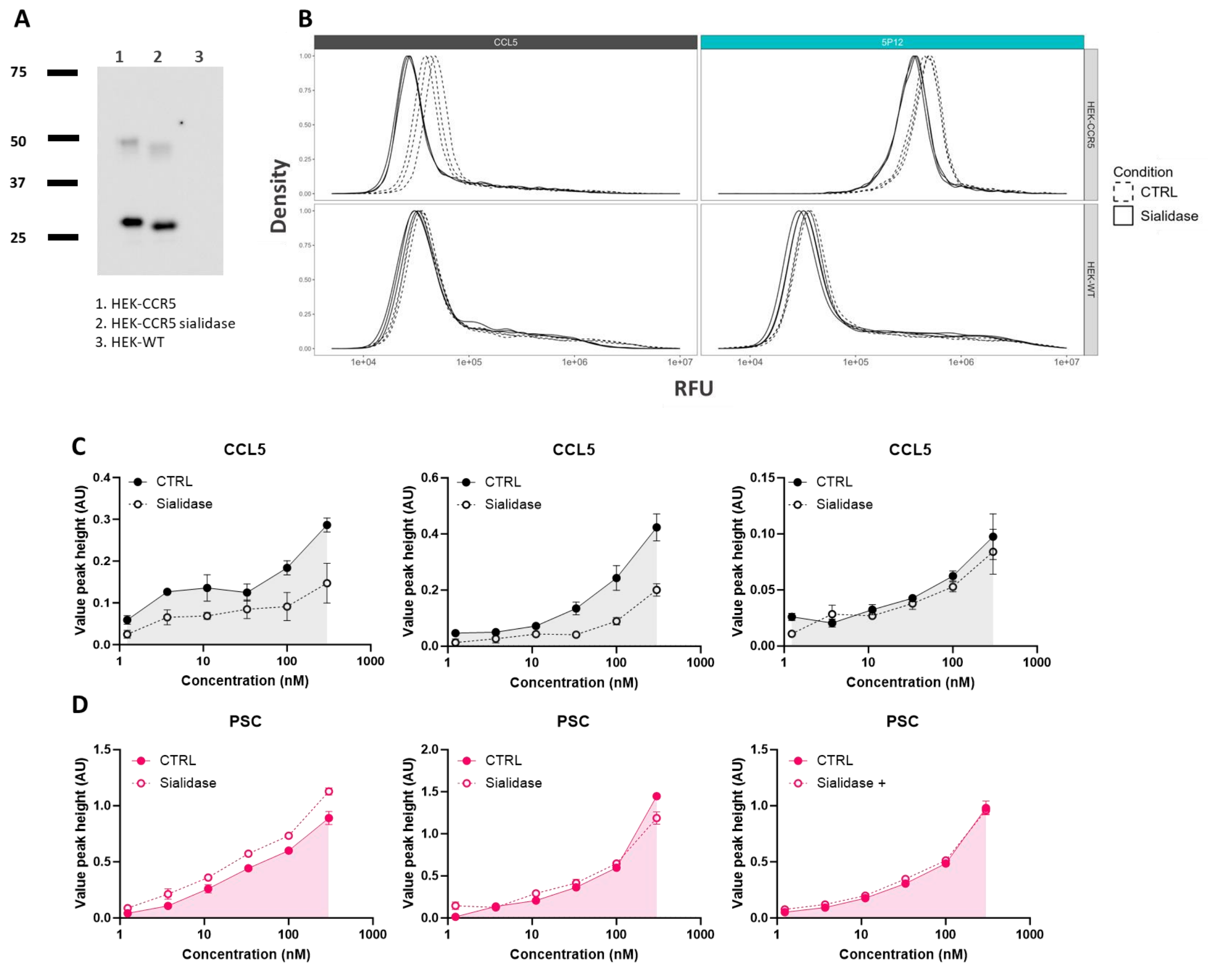


**Figure S14. Removal of sialylation from CCR5 and effects on engagement of chemokines**

**A** HEK-CCR5 and HEK-WT cells were treated with 0.3 U *Arthrobacter ureafaciens* sialidase from (Roche) or with 200 µL unsupplemented DMEM for 1.5h at 37°C and cell lysates were compared by Western Blot using the anti-CCR5 C-terminal polyclonal antibody ab63123. **B** Sialidase treated and untreated HEK-CCR5 and HEK-WT cells were incubated at 4°C for 1h with rhodamine-labelled chemokine CCL5 and 5P12-CCL5 at 300nM. Histograms of 3 independent experiments are shown. RFU = relative fluorescence units. **C, D** Sialidase treated and untreated HEK-CCR5 cells were incubated with CCL5 (**C**) or PSC-CCL5 (**D**) at the indicated concentrations and calcium flux signals were measured. Data points represent mean peak height of triplicates ± SEM and data represent 3 independent experiments.

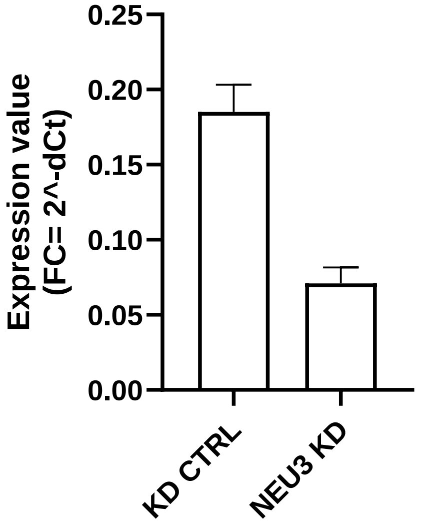


**Figure S15. Efficiency of NEU3 knockdown**

HEK CCR5 were transfected with NEU3 siRNA or negative control siRNA and mRNA expression levels of NEU3 were quantified by RTqPCR. Data represent the fold change ratio normalized to the housekeeping gene KIF11 where fold change = $2\text{^} - \mathrm{dCt}\ (\mathrm{Ct}_{\mathrm{Condition\ (NEU3\ KD\ or\ KD\ CTRL)}} - \mathrm{Average\ Ct}_{\mathrm{KIF11}})$ and represents 3 independent experiments (mean ± SEM).

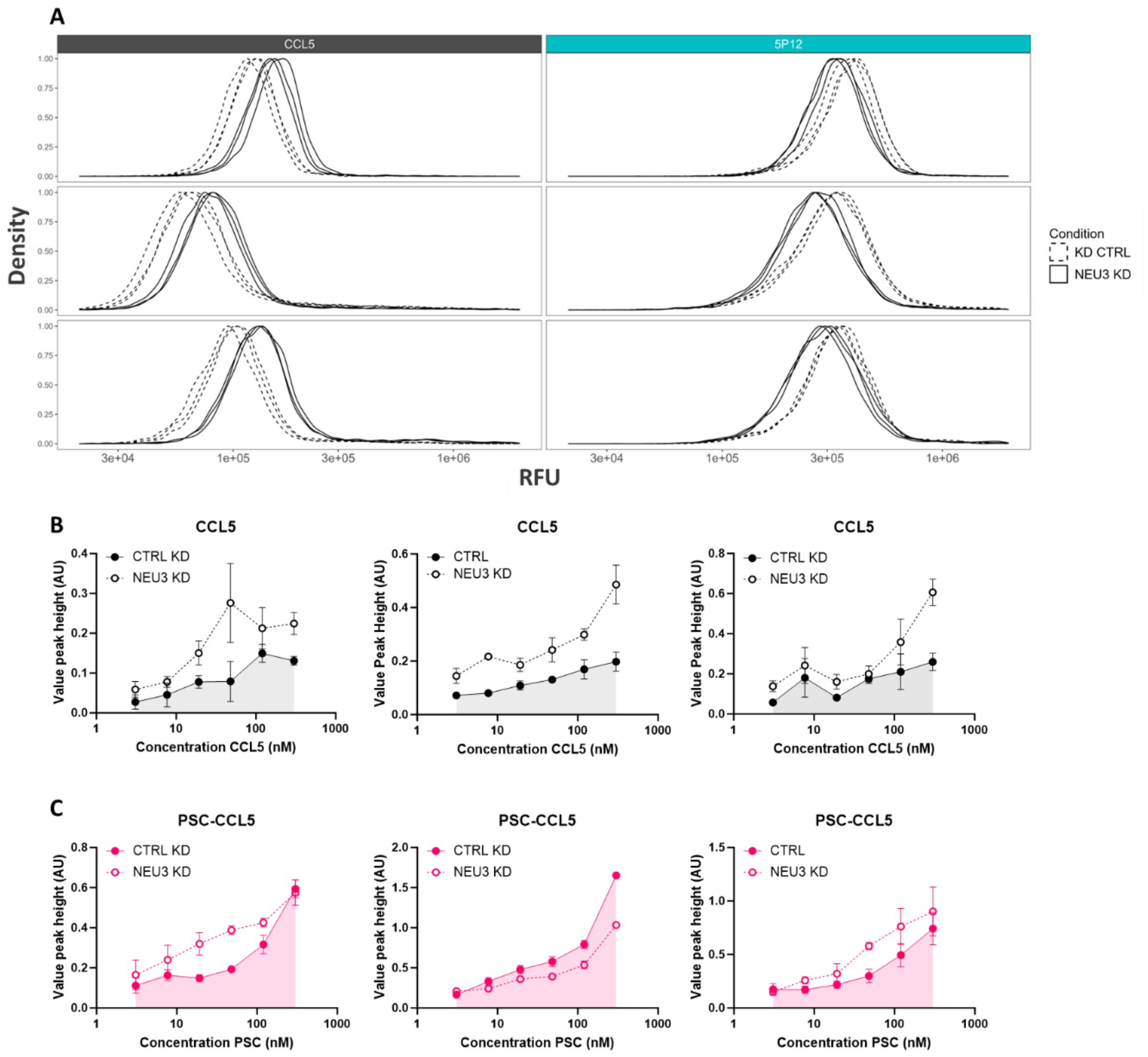


**Figure S16. Effects of NEU3 knockdown on sialylation-sensitive and sialylation-insensitive chemokines**

**A.** HEK CCR5 transfected with NEU3 siRNA (NEU3 KD) or negative control siRNA (KD CTRL) were incubated at 4°C for 1h with rhodamine-labelled chemokine CCL5 and 5P12-CCL5 at 300nM. Histograms of 3 independent experiments are shown. RFU = relative fluorescence units. **B, C** HEK-CCR5 NEU3 KD and KD CTRL cells were incubated with CCL5 (**B**) or PSC-CCL5 (**C**) at the indicated concentrations and calcium flux signals were measured. Data points represent mean peak height of triplicates ± SEM and data represent 3 independent experiments.
